# Home over institution? New insights on older adults’ care preferences from a mixed-methods study in France

**DOI:** 10.1371/journal.pone.0345491

**Published:** 2026-03-24

**Authors:** Anaïs Cheneau, Jonathan Sicsic, Thomas Rapp

**Affiliations:** 1 LIRAES and Chaire AgingUP!, Université Paris Cité, Paris, France,; 2 LIEPP (Laboratoire interdisciplinaire d’évaluation des politiques publiques), Science Po, Paris, France; U of U: University of Utah, UNITED STATES OF AMERICA

## Abstract

As populations age, long-term care policies must balance individual preferences with financial constraints. The prevailing “aging in place” policy in France assumes that citizens overwhelmingly prefer home care over nursing homes. However, little is known about people’s preferences towards long-term care options before disability occurs. We elicit preferences among community-dwelling adults over 60 using a mixed-method approach: qualitative interviews and a two-stage D-efficient discrete choice experiment. In each task, respondents chose between two hypothetical nursing homes varying in professional care quality, living environment, out-of-pocket (OOP) cost, and proximity, then decided whether to receive care in this nursing home or remain at home. A sample of 2,886 French adults over 60 completed the survey in 2024. We used random-effect conditional logit and latent class logit models to investigate trade-offs and preference heterogeneity. While a majority (54%) consistently favored home-care, 37% shifted their decision in response to improved nursing home characteristics. Professional care quality and living environment influenced choices as strongly as OOP cost, while proximity plays a secondary role. Strengthening staffing and training, upgrading equipment and the conviviality of shared spaces, and containing OOP costs are direct levers to raise the acceptability of nursing home care.

## 1. Introduction

For the past twenty years, most OECD countries have been implementing “aging in place” policies that promote home care over institutional care, shifting spending away from nursing home care and towards home care [1]. While average public allowances for institutional care declined, the share of elderly recipients receiving home care in OECD countries has risen from 59% in 2000 to 65% in 2013 and 69% in 2020 [2,3].

This trend towards deinstitutionalization and balancing long-term care (LTC) spending towards home care is not uniform across countries. For instance, while the number of beds in nursing homes or hospitals per 1,000 people aged 65 and over increased in countries such as the Netherlands, Italy, and Germany between 2011 and 2021, France experienced a sharper decline, averaging 9.7 fewer beds per 1,000 elderly individuals compared to an OECD average reduction of 4.7 beds [4].

In France, LTC is provided either at home or in a nursing home (EHPAD, *Etablissement d’hébergement pour personnes âgées dépendantes*). Public support relies primarily on the Personalized Autonomy Allowance (*Allocation personnalisée d’autonomie*, APA), a means-tested subsidy for care services adjusted to each beneficiary’s degree of disability and financial resources. In 2023, 1.36 million people aged over 60 received *APA*, including 815,800 at home and 548,960 in nursing homes [5]. Nursing-home residents tend to be older and more disabled than APA recipients living at home: in 2019, the median age at admission to an EHPAD was 88 years, and severe loss of autonomy –corresponding to individuals confined to bed or chair and/or individuals with altered mental functions requiring permanent supervision– concerned 57% of EHPAD residents, versus 20% among APA recipients living at home [5]. Severe dependency at home is therefore not exceptional, but it typically requires a combination of formal home-care services, adapted housing, and often substantial involvement of relatives, and can also be shaped by family relationships, caregiver burden, and behavioral symptoms in cognitive impairment. Beyond APA, low-income individuals may receive *Aide sociale à l’hébergement* (ASH), a means-tested subsidy that helps cover accommodation costs in nursing homes; housing benefits and tax relief may also reduce net payments. The average out-of-pocket amount in institutions is about €1,957/month among residents who do not receive ASH and €921/month among ASH recipients. By contrast, for APA beneficiaries at home, the average remaining co-payment to the APA plan is estimates at about €47€/month in 2019, although this metric captures only APA plan and does not include broader living costs or informal care [6].

Policies favoring LTC spending towards home care reflect a political strategy to contain public expenditure, as nursing home care is often more expensive than home care. For the most disabled older people, the monthly cost of dependency (expenses for support, care, and accommodation) ranges from €3,100 to €3,400 in nursing home, compared with around €2,000 to €3,000 at home, depending on the level of dependency [7]. These costs do not include informal care, which is more substantial at home. Aging-in-place policies also pursue broader objectives, including people-centered care and responsiveness to older people’s expectations. In France, two-thirds of people declare that they would prefer to avoid living in a nursing home in the future [8]. This “institutionalization aversion” [9] would reflect the disutility of nursing home care and the importance of preserving personal identity, privacy, autonomy, and social ties –values often perceived as compromised in nursing homes [10]. Yet, such aging-in-place policies rely on a strong yet largely untested assumption that nursing home care is an inferior good, i.e., it will always be substituted by home care if possible. Indeed, few preference-based studies have analyzed older people’s preferences for home care and nursing home care.

The literature exploring preferences for LTC services faces two main limitations. First, most studies that focused on the identification of preferences explored choices for home care alone [11–15] or nursing homes alone [16,17]. To our knowledge, no study has explored preferences in choosing between home and nursing home care, and no study has investigated how the characteristics of nursing homes may drive these preferences. Second, prior work focused on exploring the determinants of nursing home admission choices, such as cognitive impairments, older age, female gender, and the absence of a partner or potential informal caregivers [7,18]. As nursing home admissions are often constrained decisions, these findings do not necessarily reflect individuals’ preferences for home vs. nursing home care.

This article aims to overcome these limitations by examining the trade-off between home vs. nursing home care choices and exploring whether enhancing nursing-home facilities can actually reshape that trade-off, or whether home remains preferred regardless of nursing-home characteristics. We use a discrete choice experiment (DCE), a methodology widely used in the health economics literature, to quantify how specific nursing-home attributes (professional care quality, living environment, proximity, and out-of-pocket) shift LTC choices, by how much, and for whom. We elicit preferences among French adults aged 60 and over who are approaching LTC decisions-making but are not yet severely disabled, and ask them to make choices under scenarios of severe physical or cognitive disability. Preference heterogeneity by socioeconomic and family context is also examined, revealing responsiveness to particular levers and providing actionable guidance for LTC investment and reform.

The paper is organized as follows: Section 2 describes the methodology and DCE design. Section 3 presents the results, drawing on the DCE and qualitative interviews, and section 4 discusses implications.

## 2. Materials and methods

### 2.1 The discrete choice experiment

DCEs are a widely used stated preferences method in health economics [19] to elicit older adults’ preferences and trade-offs between care alternatives [20]. In a DCE, each alternative is described by a set of attributes (i.e., characteristics of the option, such as care quality or out-of-pocket cost). Each attribute takes one of several levels, which represent the possible values of that attribute (e.g., low/medium/high quality). Respondents are presented with repeated choice tasks in which alternatives correspond to different combination of attributes levels. Preferences are inferred from observed choices under the assumption that respondents trade off attributes when selecting their preferred option [21]. DCEs require respondents to picture a hypothetical situation in which to make choices. The framing of choice is crucial: it needs to be realistic and well understood by participants to obtain reliable answers. We defined two disability scenarios: one in which respondents were asked to imagine themselves having severe cognitive impairment and the other in which they were portrayed with severe physical impairment (S2 Appendix in S1 File provides a detailed description of each scenario). We used a between-subjects design where each respondent was randomly assigned to one scenario version throughout the questionnaire. The DCE introduced two care configurations: home care and nursing home. While intermediate residential options exist in France (e.g., *Résidences autonomie*), they are not appropriate in case of severe dependency, which justifies restricting the choice set to the two configurations most relevant under severe loss of autonomy.

**Fig 1:**
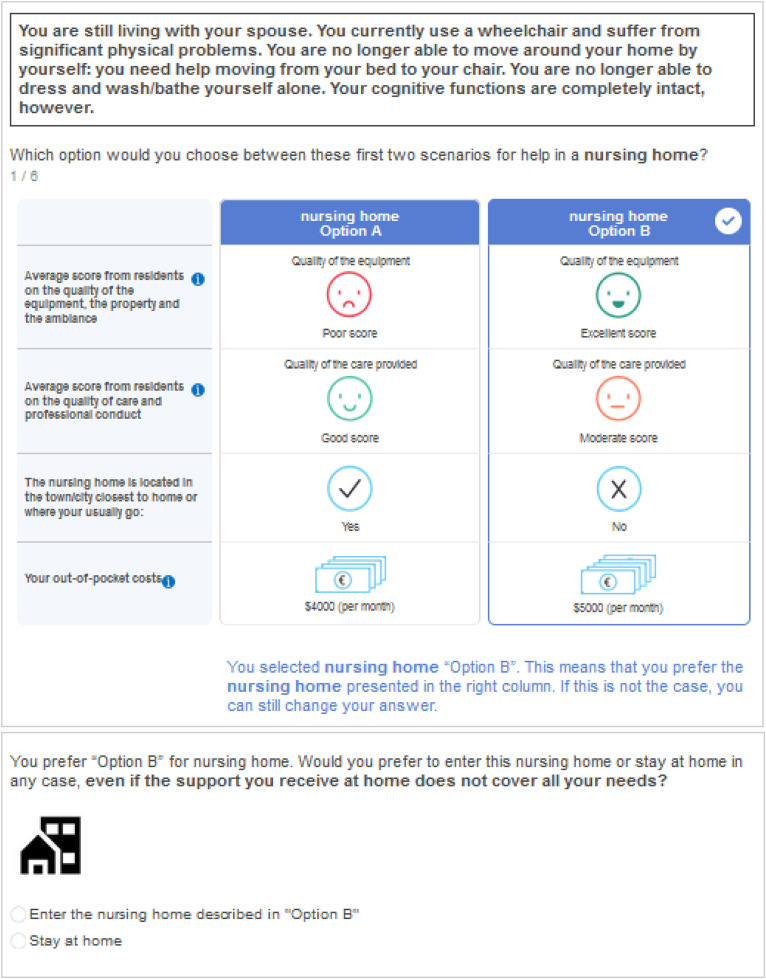
Example of a choice task. Note: The French version of the choice tasks, as seen by the respondents, is in SM B. The version shown here is a U.S.-English translation for illustration, which is why the currency symbol appears as “$” rather than “€”. The upper part of the picture reminds the respondent of the choice frame. The middle part represents the first stage choice between two competing nursing homes. The bottom part represents the second stage choice between the selected nursing home or home.

Our DCE presented respondents with several alternative bundles combining varying levels of key nursing-home attributes and asks them to select their preferred nursing-home. They completed six choice tasks involving two nursing home alternatives each (Fig 1). First, respondents chose their ‘preferred’ nursing-home among two options with varying predetermined attributes’ levels (‘forced choice’). Then, after each nursing-home task, they made an opt-out decision between the preferred nursing home and staying at home, acknowledging potential unmet needs at home. Each respondent thus made 12 choices. This two-stage format increased the amount of information gathered from choices compared to traditional pairwise choice tasks with an opt-out and allowed for modeling the drivers of opt-in vs. opt-out decisions (nursing home characteristics, contextual and respondent-specific factors). It was designed to elicit conditional preferences for nursing-home features as well as unconditional preferences for nursing home vs. home care. Importantly, this design avoided including a potentially dominant “place of living” attribute (e.g., home vs. nursing home) [22–24] known to bias choices because respondents tend to associate nursing homes with severe ill-health or end-of-life, which constrains trade-off analyses with other care arrangements attributes [24].

We hypothesized that recent scandals about severe care quality breaches (including malnutrition and unmet care needs) that involved private nursing homes in France in 2022 could have impacted respondents’ preferences regarding private nursing homes. We developed two versions of the survey that were randomly assigned to respondents: one in which the word “EHPAD” (the acronym for nursing home in France) was replaced by a more neutral but equivalent expression, “medicalized facility”. By doing so, we aimed to test for a specific aversion to the “EHPAD” among community-dwelling people.

### 2.2 Selection of attributes and levels

A DCE’s validity and relevance depend on the attributes’ selection and presentation [25–27]. We selected our attributes using two sources of information: a literature review of the results of recently published DCEs in LTC, combined with qualitative interviews. The literature review helped identify attributes commonly used to describe LTC alternatives and the range of levels considered (see details in S1 Appendix in S1 File). We used these findings to structure a semi-structured interview guide, while leaving room for respondents to introduce additional considerations (see details in S3 Appendix in S1 File). From October to December 2023, we conducted 21 interviews with people over 60 years (see S3 Appendix in S1 File for a description of the interviewed sample and the interview grid).

Using an inductive approach, we transcribed and thematically analyzed these interviews in NVivo® software [28,29]. The thematic content analysis followed an iterative process, involving identifying recurring themes in textual expressions and coding representative themes, which were progressively organized around thematic axes to build a thematic tree. Our analysis focused exclusively on verbatim references to nursing-home features that could influence the choice between home and nursing home. Four thematic attributes were identified (S3 Appendix in S1 File gathers the corresponding verbatim).

The first key attribute was the facility’s physical and social environment—rooms, common areas, shared activities—which interviewees viewed as shaping both (i) whether the setting can feel familiar and personalizes and (ii) opportunities for everyday social contact. Interviewees prioritized different aspects: food quality and menu choice, the ability to bring personal furniture, room size, access to gardens, organized activities, and friendly interactions. These features were grouped under a single attribute, “equipment, facilities, and atmosphere,” comprising five sub-dimensions: conviviality and atmosphere; size of common areas and rooms; access to gardens; feeling at home; and food quality. This attribute extends the one identified by Milte et al. (2018, 2022), which was limited to “feeling at home in their room or the shared spaces, or if residents have access to gardens” [13,14].

The second key attribute was related to professional care. Participants consistently expressed the need for reassurance about the continuous presence of staff, particularly at night, while emphasizing the importance of preserving personal autonomy and individuality. Their main concerns were the flexibility in care routines, visiting hours, meal choices, and respect for residents’ freedom and decision-making capacity. These elements were synthesized into a “quality of care and professional support” attribute, comprising seven sub-dimensions: punctuality, team size, time dedicated to care, trust-based relationships, caregiver stability, adaptability to individual needs, empathy, and professionalism. This attribute aligns with previous DCEs, highlighting the importance of the quality of professional caregivers’ services [13–17,23,24].

The third key attribute was the geographical proximity of the nursing home. Most interviewed people preferred a nursing home near or in the town where they already lived or socialized. A minority would consider relocating, but only to be closer to family for easier visits. We operationalized this as an attribute contrasting the nearest familiar town versus an unfamiliar location. To our knowledge, this proximity framing has not been examined in preference-based studies identified in our literature review (S1 Appendix in S1 File).

Finally, the fourth key attribute was the cost of LTC options. Many interviewees expressed concern that high costs –their out-of-pocket share or the financial burden on relatives– could make nursing homes unaffordable. We therefore included a monthly out-of-pocket (OOP) cost attribute, with levels ranging from €500 to €3,000, calibrated to reflect observed averages in France (around €1,850/month net of public subsidies, which exceeds older persons’ income in 75% of cases) [30].

All attributes and their levels are shown in Table 1. For the two quality attributes –(i) equipment, facilities and atmosphere and (ii) professional services and patient care–, respondents selected the three most essential subcomponents within each list: (i) conviviality and atmosphere; size of common areas and rooms; access to gardens; feeling at home; and food quality; (ii) punctuality, team size, time dedicated to care, trust-based relationships, caregiver stability, adaptability to individual needs, empathy, and professionalism. These follow-up measures let us identify which specific levers within each compound attribute drive choices, and link them to actionable investments (e.g., garden upgrades, dining services, staffing continuity, time-on-task, training in empathy, and person-centered care). All attributes were tested using think-aloud in 4 pre-pilot cognitive interviews [31]. The questionnaire was modified based on feedback from the pre-pilot interviews (S3 Appendix in S1 File details the changes made following the interviews).

**Table 1:** Attributes and levels.

Attributes presentation	Levels
The quality of the equipment, facilities and atmosphere is assessed based on the average rating given by nursing home residents each quarter: from poor rating to very good rating.	1. Poor rating 2. Medium rating 3. Good rating 4. Very good rating
The quality of professional services and patient care is assessed on the basis of the average rating given by nursing home residents each quarter: from poor rating to very good rating.	1. Poor rating 2. Medium rating 3. Good rating 4. Very good rating
The nursing home is located in the town closest to where you live or where you usually live.	0. No 1. Yes
The remaining cost you have to pay for the nursing home, e.g., what remains to be paid once public subsidies have been taken into account, varies between €500/month and €3,000/month.	1. 500€ 2. 1000€ 3. 2000€ 4. 2500€ 5. 3000€

### 2.3 Experimental design

A total of 160 combinations of attribute levels were generated in a full factorial design. We built a D-efficient design in two steps. First, we defined a fractional factorial orthogonal design with 36 alternative nursing homes randomly blocked into three questionnaire versions of six pairwise choice tasks each (2 alternatives per choice task). Second, we collected prior values from 100 respondents from a pilot survey, and used the prior derived from a multinomial logit model to build a D-efficient design of 36 unlabeled alternatives (S4 Appendix in S1 File details the priors, the 36 choice tasks, and the correlation matrix between attributes). The design was coded using the dcreate package on STATA® 2023 (Hole, 2015), which uses the modified Fedorov algorithm to optimize the search of candidate sets [32].

### 2.4 Data collection

The survey was administered online in France between July 1 and the September 29, 2024, using the quotas sampling method to obtain a representative sample according to age group, gender, region (NUTS2 –Nomenclature of territorial units of statistics, defined by Eurostat–), and income. Sampling and administration of the survey were conducted by DYNATA, an online multi-panel company. Respondents of the survey were members of Dynata’s French panelist who received an invitation to the survey and accessed the study link. At the time of filedwork, Dynata’s French panel included approximately 102,480 members aged 65 + . 83 written informed consent and received a detailed information letter providing the benefits of participating in the research, including early access to the results, and voucher incentives. Ethics approval was obtained from the University XX Ethics Committee (Project no: 2024-23-Authors). The survey included socio-demographic questions, opinions regarding home care and long-term care, behaviors and values (e.g., risk aversion, foresight, trust, anticipation of dependency, present bias, altruism, and family relationships), health status, insurance coverage, and the DCE module (at the beginning of the survey).

### 2.5 Statistical analysis

Our main dependent variable was the second stage choice between entering the selected nursing home or staying at home (see Fig 1). This outcome was binary and longitudinal, with six observations per respondent. To investigate the drivers of the nursing home vs. home care trade-offs, we estimated a random intercept conditional logit (CL) model of the probability to enter the nursing home, assuming that the decision is influenced by a random utility function U_ni_ (where *n* denoted the respondent and *i* the selected nursing home in the forced-choice task). Utility was decomposed into a deterministic utility V_nt_ and a random (unobserved) component ∈_ni_ as follow:


$$\begin{array}{cc} {\text{U}}_{\text{ni}}= & \;{\text{V}}_{\text{ni}}\;+\;\in_{\text{ni}}=\alpha_{n}+\;\beta_{\text{1}}{EQUIPMENTqual}_{ni}+\beta_{\text{2}}{CAREqual}_{ni}+\;\beta_{\text{3}}{PROXIMITY}_{ni}\\ & +\;\beta_{\text{4}}{OOP}_{ni}+\;\in_{ni}\;;\text{ n }=\;\text{1},\;\ldots .,\text{ N };\text{ i}=\text{Nursing}-\text{home A or B} \end{array}$$ (1)

where ${EQUIPMENTqual}_{ni},\ldots ,\;{OOP}_{ni}$ represented the levels of each selected nursing home *i* attributes and $\beta_{k}$ denoted each attribute’s impact (weight) in the decision, while $\alpha_{n}$ wa the random intercept, representing the respondent-specific propensity to choose to enter the nursing home over the six repeated decisions. Various Models were estimated, including the equipment and care quality rating and out-of-pocket cost, using categorical, continuous, and quadratic specifications, and the Model with the highest BIC was finally retained (S6 Appendix in S1 File details each model’s results). Categorical coding was retained in two forms: 1/ dummy coding, where each level was compared to the worst level (baseline), and 2/ stepwise coding, where each level was compared to the previous one to capture incremental effects.

We computed average marginal effects on the probability of entering the nursing home in all Models. We also calculated the attributes’ relative importance by dividing the attribute-specific level range (e.g., the difference between the highest and the lowest coefficient for the levels) by the sum of all attributes’ level ranges [33]. The final score was then multiplied by 100 for interpretation as a percentage, and attributes were ranked by order of importance on the 0–100 scale. All Models were estimated using 15,906 choice observations (2,651 respondents each making six choices) using Stata® software (xtlogit command).

In a second step, we used a latent class (LC) logit model to investigate preference heterogeneity and to identify subgroups of respondents with similar preference patterns [34,35]. The LC model was estimated using the Expectation-Maximization (EM) algorithm in the flexmix package in R, which handles estimating a finite mixture of logit models. Because the number of latent classes is not a parameter to maximize in the EM algorithm, we compared the BIC and AIC criteria across several models with varying classes ranging from 2 to 8. The four-class model was selected to achieve the highest goodness-of-fit model (BIC = 11302.67) (see S8 Appendix in S1 File for a detailed comparison of each model’s goodness of fit). Then, each respondent was allocated to a class based on their highest predicted probability, and respondents’ characteristics were compared using chi-square tests.

Note that our quantitative analysis was completed by an in-depth analysis of the qualitative material generated from the 21 audio-recorded interviews conducted before the experiment (S3 Appendix in S1 File provides details on the interviews). Verbatim explanations were coded inductively, allowing us to identify recurring motives and contextual factors influencing preferences and key decision-making rationales.

## 3. Results

### 3.1 Descriptive statistics

Of the 8,488 panel members who accessed the survey link, 3,836 met the inclusion criteria, provided informed consent, and answered the first question after consent. Among these eligible respondents, 950 dropped the survey before completing the questionnaire (25% dropouts), most often during the DCE module (83%) (see S5 Appendix in S1 File 5 for further details on the dropout sample). This observed dropout rate was within the range reported in web-based DCE studies, which commonly report dropout rate around 10–25% depending on task burden and design [36]. For the main analysis, we restricted the sample to the 2,651 respondents who answered all relevant questions—excluding those who selected “I do not want to answer”—for the variables included in the main model (i.e., age, relationship status, gender, income, housing type, rural or urban residence, number of children, experience with care, and presence of Alzheimer’s disease in the family). The average completion time was 29 minutes (median time: 20.77 minutes, minimum: 9.94 minutes).

Table 2 presents some key descriptive statistics for the sample. Of the 2,651 respondents (52.6% women), 56% were under 70, and 22% were aged 75 or over. Almost three-quarters lived with a partner (71%), and 82% had at least one child, among whom 64% lived nearby. Regarding housing, 68% lived in a house (32% in an apartment), only 21% reported fully accessible accommodations, and 24% resided in rural areas. There was a high prevalence of homeownership (76%), while 24% rented. Regarding caregiving experience, which informs about the acquaintance with care decisions, 68.5% were or had been caregivers themselves for an older relative, and 21% had or have a parent or grandparent diagnosed with Alzheimer’s disease. Most respondents had visited a relative in a nursing home before, and 28.2% never did so in the last 5 years. Regarding discrete choice patterns between nursing home and home care, 46% selected the nursing home at least once out of six choice tasks. Besides, 9.3% consistently chose the nursing home entry while 54% consistently chose to remain at home irrespective of the nursing home’s characteristics.

**Table 2:** Sample descriptive statistics.

Variable	Measurement	N = 2651	%
**Respondents characteristics**			
Sex	Female	1393	52.6
	Male	1258	47.5
Age	60-64	773	29.2
	65-69	704	26.6
	70-74	580	21.9
	75+	594	22.4
Age	Mean (SD)	69.12	(5.9)
Average salaries/ pension	Mean (SD)	2314.28	(1324.3)
Diploma	Less than high school diploma	1495	56.4
	High School Diploma or more	1156	43.6
Place of residence	Rural environment	634	23.9
	Semi-urban or urban	843	31.8
	Very dense urban	1174	44.3
In couple	Yes	776	70.7
	No	1875	29.3
Number of children	0	471	17.8
	1	441	16.6
	2	907	34.2
	3+	832	31.4
Place of living	House	1792	67.6
	Apartment in a building	859	32.4
Housing status	Homeowner	632	76.2
	Tenant or sub-tenant or free housing	2019	23.8
Adapted accommodation	Fully	549	20.6
	Partly adapted	1111	41.9
	Not at all	994	37.5
Experience in caregiving (for an older relative)	Yes	1816	68.5
	No	835	31.5
Visited a relative in a nursing home in the last 5 years	Yes	1149	43.3
	No, for a longer period	754	28.4
	No, never	748	28.2
Alzheimer’s disease in parents or grandparents	Yes	563	21.2
	No	2088	78.8
Nursing home equipment in region (from the INSEE density index)	Low level of facilities (<120 places per 1,000 elderly)	236	8.9
	Medium level (between 120 and 145 places per 1,000)	754	71.2
	Very high level (>145 places per 1,000)	528	19.9
**Discrete choice patterns**			
Choices between nursing home care and staying at home	Always nursing home	246	9.3
	Choices change	968	36.5
	*1 choice home, 5 choices nursing home*	96	3.6
	*2 choices home, 4 choices nursing home*	138	5.2
	*3 choices home, 3 choices nursing home*	190	7.2
	*4 choices home, 2 choices nursing home*	244	9.2
	*5 home choices, 1 nursing home choice*	300	11.3
	Choice: always home	1437	54.2
**Ranking of professional-care dimensions**			
Punctuality	Top 1	145	5.5
	Top 3	490	18.5
Ability to adapt to needs	Top 1	331	12.5
	Top 3	1109	41.8
Empathy and ‘*savoir-vivre’*	Top 1	544	20.5
	Top 3	1568	59.1
Time allocated for each resident	Top 1	479	18.1
	Top 3	1400	52.8
Trusting relationship	Top 1	569	21.5
	Top 3	1532	57.8
Team size	Top 1	54	2.0
	Top 3	209	7.9
Staff continuity (same professionals over time)	Top 1	428	16.1
	Top 3	1272	47.9
**Ranking of living-environment dimensions**			
Conviviality and atmosphere	Top 1	426	16.1
	Top 3	1589	59.9
Size of common areas and rooms	Top 1	377	14.2
	Top 3	1269	47.9
Access to garden	Top 1	171	6.4
	Top 3	954	35.9
Feeling ‘at-home’	Top 1	1246	47.0
	Top 3	1973	74.4
Food quality	Top 1	286	10.8
	Top 3	1670	63.0

Source: AgingUP! Survey 2024 (Chaire AgingUP!); authors’ calculations.

Finally, over a third of the sample (36,5%) changed their decision at least once and thus were responsive to variations in nursing home characteristics. In the follow-up items on what “quality” means, respondents most often ranked –on the professional care side– empathy and savoir vivre, trust-based relationships, and time spent on care among their top three elements. For the living environment, “feeling at home” was the most frequently prioritized, followed by food quality and overall conviviality and atmosphere.

### 3.2 Home vs. nursing home trade-off

#### 3.2.1 Quantitative results.

**Fig 2:**
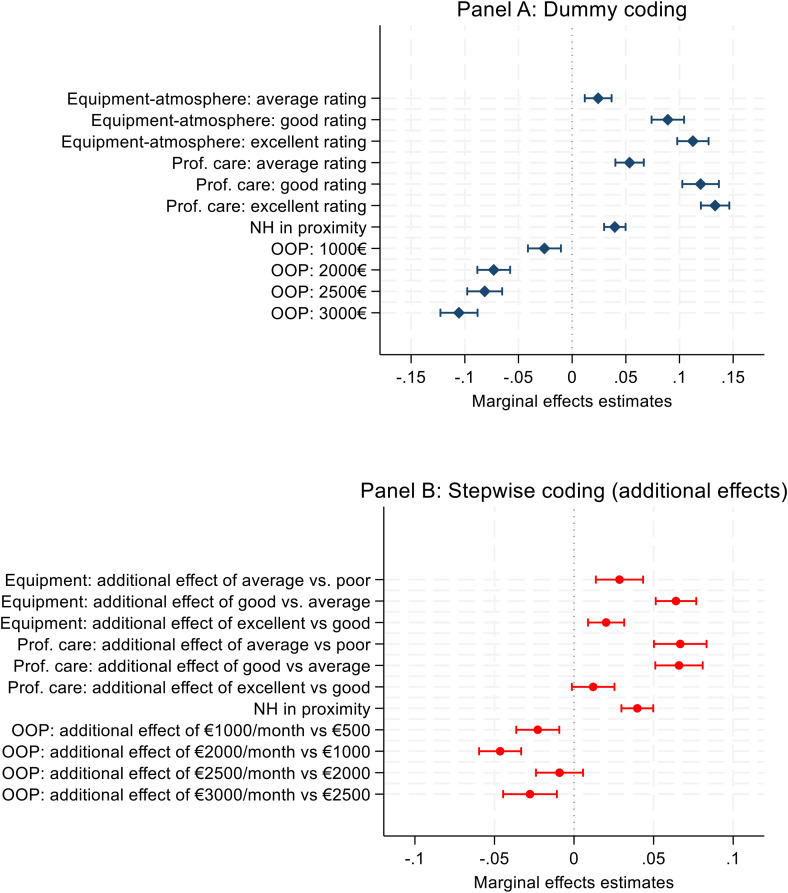
Random-effect estimations. Results of random-intercept logit model (N = 2651 individuals). The detailed coefficients are showed in SM G. In the dummy coding model (panel A), the reference categories for each attributes were poor rating (equipment-atmosphere and professional care attribute), nursing home not in closest town, and €500 out-of-pocket (OOP) cost.

The results of the conditional logit model are displayed in Fig 2 (see S7 Appendix in S1 File for coefficient estimates). First, all nursing home attributes’ levels significantly influenced the decision to choose institutional care over home care (Fig 2, panel A). The most influential attribute was the quality of care (34% of overall weight), followed by the quality of equipment (29%) and out-of-pocket expenses (27%). Geographical proximity of the nursing home also plays a role, though of lesser importance (10%).

Improving the quality of care from poor to excellent increased the probability of choosing nursing home care over home care by 13 percentage points (see S7 Appendix in S1 File for more details). A similar improvement in the quality of facilities and atmosphere increased this probability by 11 percentage points. Conversely, raising out-of-pocket expenses from €500 to €3,000 per month reduced the probability of choosing a nursing home by 10 percentage points.

Panel A of Fig 2 (dummy variable coding) shows diminishing marginal returns to quality improvements: utility gains were smaller when moving from “good” to “excellent” than from “poor” to “good” with overlapping confidence intervals. Panel B (stepwise coding) confirms that the additional effect of moving from good to excellent quality yielded smaller utility gains for the two attributes on equipment and care quality. It also reveals that the highest utility improvements came from changes from average to good quality.

To assess whether opt-out alternative is behaviorally meaningful (i.e., anchored in respondents’ circumstances rather than reflecting random choice), we re-estimated the random-intercept logit model including a broad set of individual controls (Table S8, Appendix 7 in S1 File). The marginal effects of the DCE attributes are very similar to the baseline specification without controls (Table 7 in S1 File). This suggest that conclusions regarding the relative importance or care quality, living environment and equipment, proximity and out-of-pocket costs are robust. Several individual characteristics significantly predict choices in expected directions –most notably age 75 + , female, having no recent visit in a nursing-home, and attitudes toward future disability– supporting the interpretation that opt-out choices are related to respondents’ characteristics. These individual-level pattern are examined in greater detail in the latent class analysis below.

Results of the four-class LCM (Table 3) highlight substantial preference heterogeneity for long-term care configurations and varying sensitivity to nursing home attributes. Class 1, comprising 11.5% of the sample, gathers respondents who almost always chose nursing home entry (81% always selected it, and 19% selected it on average 5 times). Within Class 2 – the largest, comprising 1,533 respondents (58% of the sample) – a vast majority (94%) systematically preferred home-care configuration, while the remaining 6% chose it nearly 5 times out of 6. These two classes were largely insensitive to nursing homes’ attribute variations; thus, conditional logit models (attributes’ impact) were not estimated for these two classes. In Classes 3 and 4, respondents are responsive to varying features of the nursing homes. Respondents in Class 3 (18% of the sample) were overall more favorable to home care (2/3 of choices). They were highly sensitive to all attributes, especially equipment quality and cost (both accounting for 64% of overall attribute importance). Therefore, institutional care was considered only under strict quality and cost conditions. Respondents in Class 4 (12% of the sample) were more favorable to a nursing home admission (selected in more than half of the time), but only when a minimum quality of professional care was ensured. For this group, care quality alone accounted for nearly half (49%) of the overall attribute’s importance. Based on these results, the four classes were qualitatively labelled: “Unconditional nursing home respondents” (Cl.1), “Unconditional home-care respondents” (Cl.2), “Home-preferring selective respondents” (Cl.3), “Minimum-care-quality driven institutionalizers” (Cl.4).

**Table 3:** Latent class estimation.

	Class 1		Class 2		Class 3		Class 4	
Labelled	Unconditional nursing home respondents		Unconditional home-care respondents		Home-preferring selective respondents		Minimum-care-quality driven institutionalizers	
Class allocation probability (%)	11.5		57.8		18.3		12.4	
Respondents (N)	304		1533		486		328	
Choice observations					(2916)		(1968)	
**Choices made**	N	%	N	%	N	%	N	%
Always nursing home (6/6 NH)	246	80.92	0	0	0	0	0	0
Varying choices -	58	19.08	96	6.26	486	100.0	328	100.0
*NH choices out of the 6 choices*	*4,98/6*		*1,02/6*		*1,86/6*		*3,3/6*	
Always home-care (0/6 NH)	0	0	1437	93.74	0	0	0	0
**Conditional logit model parameters**					Marginal effects	SE	Marginal effects	SE
Equipment rating, atmosphere (reference: poor rating)								
*Average rating*					0.133^***^	(0.021)	0.140^***^	(0.036)
*Good rating*					0.353^***^	(0.019)	0.189^***^	(0.038)
*Excellent rating*					0.490^***^	(0.028)	0.183^***^	(0.036)
Quality of care and care professionals (reference: poor rating)								
*Average rating*					0.149^***^	(0.024)	0.166^***^	(0.038)
*Good rating*					0.371^***^	(0.019)	0.315^***^	(0.040)
*Excellent rating*					0.414^***^	(0.013)	0.298^***^	(0.035)
Nursing home in proximity					0.138^***^	(0.012)	0.035	(0.026)
Out-of-pockets expenses (reference: 500€)								
*1000€*					−0.122^***^	(0.026)	−0.060^*^	(0.035)
*2000€*					−0.282^***^	(0.027)	−0.048	(0.036)
*2500€*					−0.459^***^	(0.032)	−0.042	(0.037)
*3000€*					−0.502^***^	(0.023)	−0.099^**^	(0.042)
**Attribute’s relative importance**								
Quality of equipment					32%		30%	
Quality of care and care professionals					27%		49%	
Nursing home in proximity					9%		5%	
Out-of-pockets expenses					32%		16%	

Source: AgingUP! Survey 2024 (Chaire AgingUP!); authors’ calculations.

Differences across latent classes were explored using chi-square and t-tests, and further examined through a multinomial logistic regression, using Class 2 (“Unconditional home-care respondents”) as the baseline (most dense) category (see Table 4 and S8 Appendix in S1 File for more details). “Unconditional home-care respondents” (Cl.2) were older, less likely to consider the prospect of future disability, and to have ever visited a relative in a nursing home. Compared to this class, respondents who were less averse to nursing homes (Cl.1 and Cl.4) were more likely to be males, to have higher salaries or pensions, and to have (had) a parent or grandparent with Alzheimer’s disease. Furthermore, randomization into the “medicalized facility” treatment (instead of “EHPAD”, the French widely-used acronym for nursing home) increased the likelihood of preferring a nursing home. Compared to Cl.2 respondents (reference class), “Unconditional nursing home respondents” (Cl.1) were more likely to live in an area with a minimum number of nursing home equipment, to have fewer children, a higher education degree, and to be or have been caregivers themselves. Compared to this reference class, “Minimum-care-quality driven institutionalizers” (Cl.4) were more likely to live in a flat (rather than a house), to be tenants (rather than owners), and to anticipate living older. They were less likely to be able to count on their children to help them in case of disability. Finally, compared to Cl.2, “Home-preferring selective respondents” (Cl.3) were more likely to have more children but were less likely to be able to rely on their children in the event of dependency. The couple scenario presented before each choice task (living as a couple at the time of the choice or no longer living as a couple) does not influence which of the four classes people belong to. In other words, people who are still in a couple at the time of the choice will not necessarily choose to remain at home more systematically.

**Table 4:** Description of respondents’ profiles, by latent classes.

	Cl.1	Cl.2	Cl.3	Cl.4	1vs2	3vs2	4vs2
	%				P-value differences test		
Female N, (%)	45.39	55.25	52.06	47.26	0.002	0.218	0.008
Mean Age (SD)	68.75	69.63	68.16	68.51	0.000	0.000	0.000
Average salaries or pension	2471.38	2266.24	2279.12	2445.27	0.000	0.643	0.000
Real estate and financial assets, N, (%)					0.648	0.467	0.377
*<100 000€*	40.74	39.25	37.26	42.03			
*>= 100 000€*	59.26	60.75	62.74	57.93			
Diploma, N, (%)					0.056	0.215	0.478
*Less than/or high school diploma*	51.97	57.93	54.73	55.79			
*Higher than high School Diploma*	48.03	42.07	45.27	44.21			
Number of children, mean (SD), ttest	1.78	1.91	1.98	1.91	0.000	0.005	0.796
Having at least one child living nearby, N, (%)	52.63	52.45	53.50	52.74	0.953	0.686	0.922
Live alone (DCE scenario) N (%)	66.45	64.06	64.81	62.50	0.426	0.761	0.594
Scenario physical problems (vs cognitive problems)	51.64	49.12	49.59	50.30	0.421	0.857	0.697
Nursing home equipment in region*, N, (%)					0.021	0.363	0.197
*Low levels*	4.93	9.92	8.23	8.84			
*Medium levels*	74.01	69.47	72.63	74.39			
*Very high levels*	21.05	20.61	19.14	16.77			
Living in a very dense urban area, N, (%)	21.05	20.61	19.14	16.77	0.101	0.948	0.260
Living in a house (vs. living in a flat), N, (%)	65.79	68.43	69.34	62.80	0.368	0.705	0.049
Owner (vs tenant), N, (%)	75.99	77.50	76.13	70.12	0.567	0.533	0.004
Past or present experience: caring for the elderly	76.64	68.04	64.40	62.21	0.003	0.137	0.679
Alzheimer’s disease in the family	27.96	19.05	21.60	24.70	0.000	0.216	0.020
Residential facilities for dependent people version (vs « EHPAD »)	53.95	47.29	47.74	54.27	0.034	0.864	0.022
75% chance of living to age 85 or over	40.79	39.47	37.86	46.95	0.666	0.527	0.012
Housing not at all adapted to the disabled	39.47	37.05	38.89	35.67	0.425	0.466	0.638
Have helped other family members in the past, or help them often or very often (looking after grandchildren, etc.).	55.92	53.29	49.18	49.39	0.401	0.113	0.199
Consider the possibility of one day becoming disabled	84.21	71.95	77.37	82.32	0.000	0.019	0.000
Preference for private rather than public nursing homes	44.41	44.10	41.77	46.04	0.920	0.367	0.521
Can count on children to help in case of disability	5.92	8.41	5.76	5.18	0.143	0.057	0.048
Has visited a relative in a nursing home in the last 5 years	50.99	39.86	44.65	50.61	0.000	0.061	0.000
Perceived health					0.717	0.145	0.489
*Good or very good*	67.11	69.17	64.61	70.12			
*Medium or bad*	32.89	31.83	35.39	29.88			
Chronic pain	48.68	49.38	51.44	47.56	0.824	0.429	0.550
Physical limitations	31.91	31.331	31.69	31.40	0.383	0.876	0.974

Note: *Insee density index: 1. Very high level of nursing home facilities (> 145 places per 1,000 elderly people); 2. Medium level of facilities (between 120 and 145 places per 1,000); 3. Low level of facilities (< 120 places per 1,000). Source: AgingUP! Survey 2024 (Chaire AgingUP!); authors’ calculations.

#### 3.2.2 Comparison between quantitative and qualitative results.

***Socio-demographic context and family support.*** The interviews shed light on the role of children and spouses in care arrangement trade-offs. Although our DCE results suggested that the number of children or their proximity had limited influence on stated choices, respondents’ narratives pointed to a more nuanced role of family. Many were reluctant to rely on their children for daily care. They feared burdening their children, who often combined work and family life with childcare responsibilities. Occasional support (e.g., shopping or administrative help) was viewed as acceptable but respondents generally did not see children as substitutes for professional care when needs became intensive or involve personal care. At the same time having children nearby was often valued for emotional support and social contact, as it could reduce loneliness and thereby reinforce the appeal of remaining at home.

“But afterwards, I’d go into a nursing home, I’m not going to bother my daughter” (PAF13)

[Could you count on your son to help you if needed?]” Every day I couldn’t, no, that’s not possible, he wouldn’t be able to do his job or anything. I’m here, he has four children, he has a wife, well, it’s impossible.” (PAF15)

“They’re very present [her children], they’ll probably be there to help me if I have a computer problem or if I need groceries. But they won’t be able to come and help me every day” (PAF14)

“We’re indeed isolated; we’re not in town for shopping, but we still have our son XX, who lives nearby now and can always help us, do the shopping. It’s not like we’re all alone, completely alone.”(PAF02)

The presence of a spouse also played a crucial role, while not being a significant factor in quantitative analyses. Couples often acted as mutual caregivers and provided emotional support, making home care more feasible. Respondents indicated that their preferences would likely shift after the death or health decline of a partner.

“I think we’ll decide when one of us is gone. The last one standing will make the decision. As long as we have two, we’ll stay home. When there are two of us, there’s always one who’s in better shape than the other. So I think we’d still be better off at home. And above all, when two of us exist, we’re not isolated.” (PAF02)

“The real problem is if my wife’s illness gets worse, it’s going to cause problems. I’ll be here alone the day she has to go to a specialized institute. It’s also going to cost a lot of money. I could handle it, but that means I need someone else to help me a lot more. Or else, I’ll be the one to leave. She won’t stay here alone, she can’t.” (PAH09)

***Health status.*** Quantitative results showed that expecting future limitations or living to an old age increased the likelihood of choosing a nursing home, whereas the type of limitation presented in the hypothetical scenarios (cognitive vs. physical) did not significantly shape the preference. One likely explanation is that both scenarios described severe dependency, leaving limited room for respondents to differentiate between impairment types. Interviews supported this result, highlighting that the severity of disability (e.g., being bedridden, falling frequently, or not being able to wash themselves or get dressed alone) was the main driver of institutionalization decision, rather than whether limitations were cognitive or physical.

“If the children are worried that I’ll get lost somewhere or forget to turn off the gas, then yes, I’ll think about it [the nursing home solution].” (PAF01)

“I think you really have to be bedridden to be in a nursing home. If you can’t move, you can’t do anything. Yes, of course”. (PAF06)

“It’s a solution [nursing homes] that is indeed possible when you are extremely dependent, when you need help to wash yourself. I think at some point, there’s no choice.” (PAF14).

***Familiarity with the LTC system.*** Quantitative results showed that caregiving experience, experience in visiting relatives in nursing homes, or experience with Alzheimer’s disease within the family increased the likelihood of considering nursing home entry. During the interviews, respondents often referred to a relative in a nursing home to justify the positive and/or negative aspects of nursing homes. Experience in caregiving seems to play an important role, although it is difficult to disentangle a generational or experience effect as a caregiver. One of the interviewees was herself a caregiver for her mother after she suffered a fractured femoral neck. She felt it was natural to help her mother and to do everything she could to prevent her from going into a nursing home. In contrast, for herself, she would prefer to go into a nursing home and not be a burden on her children.

“It never occurred to me to move her [her mother] to a nursing home. [...] For me, yes, I don’t want to be a burden on my children in general. I don’t want to have to rely on them.” (PAF10)

***Representations and characteristics of nursing homes.*** All included nursing home characteristics played a key role in shaping preferences, though not all respondents reacted to variations in the selected attributes. Qualitative work supports this result by revealing that changing one or two nursing home attributes cannot alter decisions. There is a considerable effort to be made to improve the way nursing homes are represented and to reinvent nursing homes that are more humane and attractive. The most common criticisms were the depressing atmosphere, lack of social engagement, and concentration of highly dependent individuals. Many rejected the idea of entering a nursing home as they are currently known, even if they were open to other forms of shared living with peers. Quantitative results supported the assumption that using the acronym “EHPAD” rather than “medicalized facility” generated greater fear and suspicion among respondents. Qualitative work suggested smaller facilities, where people can participate in community life and where individual freedoms are respected (eating when you want, etc.).

“The nursing home here is old-fashioned. It hasn’t changed since my mother went there. Even today, there are still rooms for two.” (PAF07)

“I don’t see myself surrounded by elderly people who are worse than me, even like me.” (PAF04)

“When you arrive [at a nursing home] and you see them all lined up in a row, I have this image in front of my eyes, frankly, and... it’s not a happy sight.” (PAF13)

“I have a friend who lives in a house with about 8-10 other people, and they all share their lives and cook together. She brought her furniture into her room. It’s something I could consider” (PAF02)

“I want to be free, independent. If I feel like eating at 2 p.m., I eat at 2 p.m.” (PAF11)

## 4. Discussion

In the context of population ageing, this study provides new evidence on LTC services preferences among French community-dwelling adults aged 60 and over. While the dominant policy narrative in France assumes a strong preference for ageing at home, our findings reveal a more nuanced picture. In situations of severe dependency, as depicted in our DCE, nearly half of the respondents prefer entering a nursing home at least once across six different situations, especially when key quality and affordability criteria are met. This underscores the need to move beyond a simplistic home vs. institution debate. Targeted improvements in nursing home environments could make institutional care a more acceptable option for many older adults.

Moreover, we find significant preference heterogeneity and document the key drivers underpinning care mode choices. Individuals less averse to nursing homes tend to be younger, male, and expect to live longer. In contrast, strong aversion to nursing homes appears associated with limited exposure and knowledge of LTC: individuals who have never visited a nursing home, who struggle to envision their dependency, or who do not have experience with illness and caregiving. Among those with a stronger preference for nursing homes, we identify two distinct profiles: one consistently prefers institutional care, likely due to the absence of daily support (e.g., single men) or to avoid burdening family members, especially when they have past caregiving experience and anticipate a long life. The other group exhibits conditional preferences, influenced by nursing home characteristics. In our sample, this profile represents 36.5% of respondents. This group, composed primarily of higher-income male tenants, appears very responsive to policy interventions to improve the sector. In particular, improvements in professional care quality (especially the relational dimension of care) and in equipment and amenities (especially more home-like environments) substantially increase the likelihood that these respondents consider nursing homes as an acceptable option.

Our study faces three main limitations. First, the results are based on stated preferences elicited through hypothetical scenarios, which may be subject to hypothetical bias and may not accurately predict the real-life choices of respondents. We followed state-of-the-art practices to reduce the risk of hypothetical bias. Our extensive preliminary qualitative work combining semi-structured interviews with cognitive interviews (using think-aloud) ensured that all attributes were understood and relevant, thus enhancing the face validity of our approach [37]. Nonetheless, it remains challenging for respondents who are not yet severely disabled to anticipate preferences under severe dependency. Present bias optimism bias, or misperceptions about future needs and affordability may have influenced choices, and the direction of any resulting bias cannot be determined.

Second, using an online survey panel may introduce a selection bias. Whilst the sample is broadly comparable to the French population aged 60 and over in terms of age distribution, gender, income, and region, we cannot rule out self-selection regarding other unobserved characteristics. Indeed, online surveys require respondents to be computer-literate and may thus overrepresent individuals with higher socioeconomic status. Latent class model results allow understanding how various socio-economic characteristics shape preferences for care arrangements.

Third, our design involved a trade-off between realism and cognitive burden. In principle, DCEs can incorporate alternative-specific attributes and directly compare options such as home care and nursing homes. In practice, however, when alternatives differ on many dimensions, making both profiles equally detailed (e.g., specifying type and intensity of home care services, coordination, costs, home adaptations, etc.) rapidly inflates task complexity and degrades response quality. Our pre-tests showed that richer home-care descriptions substantially increased cognitive burden for respondents. We therefore opted to keep the nursing-home attributes detailed while modeling the home option’s baseline utility with respondent background (housing type, urban/rural, disability-adapted housing) and treating “staying at home” as an opt-out after each nursing-home choice. To mitigate what remains unobserved or more holistic (symbolic meanings of “home” or “institutions”), we complemented the DCE with semi-structured interviews to elicit decision logics that cannot be modeled without excessive task complexity. Even with this design, the DCE remained cognitively demanding. Notably, 83% of respondents who dropped the survey did so during the DCE module, with attribution somewhat higher among older respondents and those with fewer economic resources (see S5 Appendix 5 in S1 File for further details on the dropout sample). This attrition is unlikely to have compromised the representativeness of the final sample, as quota targets for sex, age, income and region were met. Still, each choice task required respondents to simultaneously project themselves into a situation of severe dependency, a future household context, and the care alternatives described in the DCE. This cognitive load may have reduced respondent’s ability to incorporate some contextual factors–such as couple life situation or the limitation type (cognitive versus physical)– thereby contributing to their limited influence on the stated trade-off between home-care and nursing-home care, despite their prominence in studies of observed institutionalization trajectories and determinants of home-care use [20,38,39].

Our results have several policy implications. Public investment should focus on what people value most, particularly for those whose choices are responsive to nursing-home characteristics, representing 36.5% of our sample. On care quality first, investments should prioritize training more professionals in interpersonal skills –empathy, savoir-vivre, trust-building– and allocating more time per resident. Then, on the living environment, improving food quality and upgrading rooms and commons areas (furnishings, building design) could create a more “home-like”, convivial setting. These concrete features shift preferences toward institutional care. Reducing out-of-pocket expenses and ensuring territorial equity in facility availability also appear to be effective in attracting the elderly, aligning with prior findings [40,38]. Such improvements could lead more than one third of older adults (36.5% in our sample) to choose nursing-home rather than home care when faced with severe dependency. This suggest that aversion to institutional care is, for many, not fixed but conditional on quality and daily-life conditions.

Our results support the idea that nursing homes should continue to fulfill essential roles and could become more acceptable and desirable options through public investment. Recent studies point the same way: most adverse effects associated with nursing home entry (e.g., loneliness, depression, anxiety) are usually temporary and concentrated in the initial months [39]; and nursing homes may prevent specific risk factors associated with mortality, such as cognitive decline and Alzheimer’s disease [41]. By contrast, substituting nursing home care for home care increases the risk of hospital admission, intensifies burdens on informal caregivers, and fails to generate cost savings for the state [42,43]. These considerations reinforce the need for targeted investment in the nursing home sector.

## Supporting information

S1 File **S1 Appendix**: Literature review for disabled persons and LTC configurations. **S2 Appendix**: Scenario and choice tasks presentation. Figure S2: Choice tasks presentation. **S3 Appendix**: Qualitative phase. Table S3.A: Characteristics of people surveyed for the qualitative stage. Table S3.B: Interview grid. Table S3.C: Thematic tree of interviewees’ expectations for nursing homes. **S4 Appendix**: Experimental design. Table S4.A: Matrice correlation for nursing home design. Table S4.B: 36 choice sets of nursing home DCE. **S5 Appendix**: Data sample. Table S5.A: Age group target quotas. Note: *Eurostat (2023).* Table S5.B: Regions target quotas. Note: Eurostat (2023). * « Régions Ultrapériphériques françaises »: Guadeloupe, Martinique, France Guyane, La Réunion, Mayotte, Saint-Martin. Table S5.C: Income target quotas. Notes: *INSEE data (admin data, public and private sector combined; no age criteria): 1st decile = 1440; 1st quartile = 1680; Median = 2095; 3rd quartile = 2765; 9th decile = 3765. Furthermore, according to the 2010 Wealth Survey, median salary levels are not significantly different between those under and over 50. However, salaries fall slightly from the age of 65.* Table S5.D: Dropout sample. Note: *The dropout sample includes individuals who met the inclusion criteria, provided informed consent, and answered the first survey question after consent, but discontinued the survey before completing the questionnaire. **S6 Appendix:** Specifications tests. Note: Standard errors in parentheses. * p < 0.10, ** p < 0.05, *** p < 0.01. **S7 Appendix**: DCE Results. Table S7.A: Random-intercept logit model. Note: Coefficient; Standard errors in parentheses. * p < 0.10, ** p < 0.05, *** p < 0.01. Table S7.B: Random-intercept logit model with individual controls. Note: Coefficient; Standard errors in parentheses. * p < 0.10, ** p < 0.05, *** p < 0.01. **S8 Appendix**: Latent class logit models. Table S9.A: Model goodness of fit results. Note: *AIC = Akaike information criterion; BIC = Bayesian information criterion.* Table S9.B: Latent class estimation. *Table note: Values are shown for each latent class as n (%) for categorical variables and mean (SD) for continuous variables. The columns labeled “1 vs 2”, “1 vs 3”, “1 vs 4”, “2 vs 3”, “2 vs 4”, and “3 vs 4” report pairwise p-values for differences between classes (χ² tests for categorical variables; two-sample t-tests for continuous variables). For example, the proportion of women was 45.42% in Class 1 and 55.45% in Class 2, and this difference was statistically significant (p < 0.001).* Table S9.C: Multinomial logit. Note: Standard errors in parentheses. * p < 0.10, ** p < 0.05, *** p < 0.01.
(DOCX)

S2 File Codebook. (PDF)

S1 Data Data. (XLSX)

## References

[1] Lee S-H, Chon Y, Kim Y-Y. Comparative Analysis of Long-Term Care in OECD Countries: Focusing on Long-Term Care Financing Type. Healthcare (Basel). 2023;11(2):206. doi: 10.3390/healthcare11020206 36673574 PMC9858923

[2] Colombo F, Llena-Nozal A, Mercier J, Tjadens F. Help Wanted?: Providing and Paying for Long-Term Care [Internet]. OECD; 2011 [cited 2025 Jan 29]. (OECD Health Policy Studies). Available from: https://www.oecd.org/en/publications/help-wanted_9789264097759-en.html

[3] OECD. Health at a Glance 2015 OECD Indicators [Internet]. OECD Publishing; 2015. (Health at a Glance). Available from: https://books.google.fr/books?id=62OQDwAAQBAJ

[4] OECD. Health at a Glance 2023: OECD Indicators [Internet]. OECD; 2023 [cited 2025 Apr 4]. (Health at a Glance). Available from: https://www.oecd.org/en/publications/health-at-a-glance-2023_7a7afb35-en.html

[5] Le Caignec É. L’aide sociale aux personnes âgées ou handicapées - Edition 2025. Panorama de la DREES; 2025.

[6] Boneschi S, Miron de L’Espinay A. Aides à l’autonomie des personnes âgées: qui paie quoi? - L’apport du modèle Autonomix – résultats 2019. Les dossiers de la DREES. 2022;(99).

[7] Gramain A, Roquebert Q, Tenand M. Aide informelle à domicile et en EHPAD: déterminants, valeur monétaire et implication pour la répartition des coÛts de la dépendance. Revue d’économie financière. 2023;152(4):125–39.

[8] Antunez K. Les Français souhaitent une prise en charge par l’État de la perte d’autonomie des personnes âgées. Etudes et Résultats. 2020;1148(1148).

[9] Costa-Font J. “Institutionalization aversion” and the willingness to pay for home health care. Journal of Housing Economics. 2017;38:62–9. doi: 10.1016/j.jhe.2017.10.001

[10] Organisation mondiale de la Santé. Rapport mondial sur le vieillissement et la santé [Internet]. Genève: Organisation mondiale de la Santé; 2016 [cited 2025 Jan 31]. 279 p. Available from: https://iris.who.int/handle/10665/206556

[11] Amilon A, Ladenburg J, Siren A, Vernstrøm Østergaard S. Willingness to pay for long-term home care services: Evidence from a stated preferences analysis. The Journal of the Economics of Ageing. 2020;17:100238. doi: 10.1016/j.jeoa.2020.100238

[12] de Bresser J, Knoef M, van Ooijen R. Preferences for in-kind and in-cash home care insurance. J Health Econ. 2022;84:102626. doi: 10.1016/j.jhealeco.2022.102626 35569208

[13] Kaambwa B, Lancsar E, McCaffrey N, Chen G, Gill L, Cameron ID, et al. Investigating consumers’ and informal carers’ views and preferences for consumer directed care: A discrete choice experiment. Soc Sci Med. 2015;140:81–94. doi: 10.1016/j.socscimed.2015.06.034 26210656

[14] Lehnert T, Günther OH, Hajek A, Riedel-Heller SG, König HH. Preferences for home- and community-based long-term care services in Germany: a discrete choice experiment. Eur J Health Econ. 2018;19(9):1213–23. doi: 10.1007/s10198-018-0968-0 29626266

[15] Walsh S, O’Shea E, Pierse T, Kennelly B, Keogh F, Doherty E. Public preferences for home care services for people with dementia: A discrete choice experiment on personhood. Soc Sci Med. 2020;245:112675. doi: 10.1016/j.socscimed.2019.112675 31760321

[16] Milte R, Ratcliffe J, Chen G, Crotty M. What Characteristics of Nursing Homes Are Most Valued by Consumers? A Discrete Choice Experiment with Residents and Family Members. Value Health. 2018;21(7):843–9. doi: 10.1016/j.jval.2017.11.004 30005757

[17] Milte RK, Mpundu-Kaambwa C, Chen G, Crotty M, Ratcliffe J. What constitutes preferred long-term care provided in residential aged care facilities? An empirical comparison of the preferences of the general population, residents, and family members. Value Health. 2022;25(2):257–67. 35094799 10.1016/j.jval.2021.09.001

[18] Luppa M, Luck T, Weyerer S, König H-H, Brähler E, Riedel-Heller SG. Prediction of institutionalization in the elderly. A systematic review. Age Ageing. 2010;39(1):31–8. doi: 10.1093/ageing/afp202 19934075

[19] Clark MD, Determann D, Petrou S, Moro D, de Bekker-Grob EW. Discrete choice experiments in health economics: a review of the literature. Pharmacoeconomics. 2014;32(9):883–902. doi: 10.1007/s40273-014-0170-x 25005924

[20] Mentzakis E, Ryan M, McNamee P. Using discrete choice experiments to value informal care tasks: exploring preference heterogeneity. Health Econ. 2011;20(8):930–44. doi: 10.1002/hec.1656 20799343

[21] Rapp T, Cheneau A, Kingsada A, Sicsic J. L’analyse des préférences individuelles pour améliorer l’efficience des innovations pour la prévention en santé. Innovations & Prévention en Santé. LEH Edition. 2025.

[22] Dixon S, Nancarrow SA, Enderby PM, Moran AM, Parker SG. Assessing patient preferences for the delivery of different community-based models of care using a discrete choice experiment. Health Expect. 2015;18(5):1204–14. doi: 10.1111/hex.12096 23809234 PMC5060844

[23] Leng A, Liu J, Maitland E, Li S, Nicholas S, Ma B, et al. Older adults preferences for long-term caregivers in China: a discrete choice experiment. Public Health. 2024;231:158–65. doi: 10.1016/j.puhe.2024.03.022 38692091

[24] Nieboer AP, Koolman X, Stolk EA. Preferences for long-term care services: willingness to pay estimates derived from a discrete choice experiment. Soc Sci Med. 2010;70(9):1317–25. doi: 10.1016/j.socscimed.2009.12.027 20167406

[25] Coast J, Al-Janabi H, Sutton EJ, Horrocks SA, Vosper AJ, Swancutt DR, et al. Using qualitative methods for attribute development for discrete choice experiments: issues and recommendations. Health Econ. 2012;21(6):730–41. doi: 10.1002/hec.1739 21557381

[26] DeShazo JR, Fermo G. Designing Choice Sets for Stated Preference Methods: The Effects of Complexity on Choice Consistency. Journal of Environmental Economics and Management. 2002;44(1):123–43. doi: 10.1006/jeem.2001.1199

[27] Lancsar E, Louviere J. Conducting discrete choice experiments to inform healthcare decision making: a user’s guide. Pharmacoeconomics. 2008;26(8):661–77. doi: 10.2165/00019053-200826080-00004 18620460

[28] Campbell JA, Ezzy D, Neil A, Hensher M, Venn A, Sharman MJ, et al. A qualitative investigation of the health economic impacts of bariatric surgery for obesity and implications for improved practice in health economics. Health Econ. 2018;27(8):1300–18. doi: 10.1002/hec.3776 29855095

[29] Hughes N, Locock L, Ziebland S. Personal identity and the role of “carer” among relatives and friends of people with multiple sclerosis. Soc Sci Med. 2013;96:78–85. doi: 10.1016/j.socscimed.2013.07.023 24034954 PMC3778435

[30] Barber SL, Van Gool K, Wise S, Woods M, On Z, Penneau A, et al. Pricing long-term care for older persons. WHO Center for Health Development, OECD. 2021.

[31] Ryan M, Watson V, Entwistle V. Rationalising the “irrational”: a think aloud study of discrete choice experiment responses. Health Econ. 2009;18(3):321–36. doi: 10.1002/hec.1369 18651601

[32] Carlsson F, Martinsson P. Design techniques for stated preference methods in health economics. Health Econ. 2003;12(4):281–94. doi: 10.1002/hec.729 12652515

[33] Bien DR, Danner M, Vennedey V, Civello D, Evers SM, Hiligsmann M. Patients’ Preferences for Outcome, Process and Cost Attributes in Cancer Treatment: A Systematic Review of Discrete Choice Experiments. Patient. 2017;10(5):553–65. 28364387 10.1007/s40271-017-0235-y PMC5605613

[34] Greene WH, Hensher DA. A latent class model for discrete choice analysis: contrasts with mixed logit. Transportation Research Part B: Methodological. 2003;37(8):681–98. doi: 10.1016/s0191-2615(02)00046-2

[35] Hess S. Latent class structures: taste heterogeneity and beyond. In: Hess S, Daly A, editors. Handbook of Choice Modelling [Internet]. Edward Elgar Publishing; 2014 [cited 2025 June 13]. Available from: https://china.elgaronline.com/view/edcoll/9781781003145/9781781003145.00021.xml

[36] Veldwijk J, Johansson JV, Donkers B, de Bekker-Grob EW. Mimicking Real-Life Decision Making in Health: Allowing Respondents Time to Think in a Discrete Choice Experiment. Value Health. 2020;23(7):945–52. doi: 10.1016/j.jval.2020.02.014 32762997

[37] Johnston RJ, Boyle KJ, Adamowicz W (Vic), Bennett J, Brouwer R, Cameron TA, et al. Contemporary Guidance for Stated Preference Studies. Journal of the Association of Environmental and Resource Economists. 2017;4(2):319–405. doi: 10.1086/691697

[38] Mommaerts C. Are coresidence and nursing homes substitutes? Evidence from Medicaid spend-down provisions. J Health Econ. 2018;59:125–38. doi: 10.1016/j.jhealeco.2018.04.003 29709710 PMC5966342

[39] Bom J, Bakx P, Rellstab S. Well-being right before and after a permanent nursing home admission. Health Econ. 2022;31(12):2558–74. doi: 10.1002/hec.4595 36057846 PMC9826495

[40] Carrère A, Jusot F. Modes de prise en charge de la perte d’autonomie: l’offre contraint-elle les choix des personnes âgées?. Revue économique. 2020;71(6):1069–99.

[41] Carrère, A, Roy, D, Toulemon, L. Vieillir à domicile: disparités territoriales, enjeux et perspectives. Rapport de l’IPP. 2023;41(41).

[42] Bakx P, Wouterse B, van Doorslaer E, Wong A. Better off at home? Effects of nursing home eligibility on costs, hospitalizations and survival. J Health Econ. 2020;73:102354. doi: 10.1016/j.jhealeco.2020.102354 32663638

[43] Pickard L. A growing care gap? The supply of unpaid care for older people by their adult children in England to 2032. Ageing and Society. 2015;35(1):96–123.

