# Supplementary material for: Home over institution? New insights on older adults’ care preferences from a mixed-methods study in France
Source: PLoS One. 2026 Mar 24;21(3):e0345491. doi: 10.1371/journal.pone.0345491 (PMC13012468; doi:10.1371/journal.pone.0345491)
Supplement: S1 File — S1 Appendix: Literature review for disabled persons and LTC configurations. S2 Appendix: Scenario and choice tasks presentation. Figure S2: Choice tasks presentation. S3 Appendix: Qualitative phase. Table S3.A: Characteristics of people surveyed for the qualitative stage. Table S3.B: Interview grid. Table S3.C: Thematic tree of interviewees’ expectations for nursing homes. S4 Appendix: Experimental design. Table S4.A: Matrice correlation for nursing home design. Table S4.B: 36 choice sets of nursing home DCE. S5 Appendix: Data sample. Table S5.A: Age group target quotas. Note: Eurostat (2023). Table S5.B: Regions target quotas. Note: Eurostat (2023). * « Régions Ultrapériphériques françaises »: Guadeloupe, Martinique, France Guyane, La Réunion, Mayotte, Saint-Martin. Table S5.C: Income target quotas. Notes: INSEE data (admin data, public and private sector combined; no age criteria): 1st decile = 1440; 1st quartile = 1680; Median = 2095; 3rd quartile = 2765; 9th decile = 3765. Furthermore, according to the 2010 Wealth Survey, median salary levels are not significantly different between those under and over 50. However, salaries fall slightly from the age of 65. Table S5.D: Dropout sample. Note: *The dropout sample includes individuals who met the inclusion criteria, provided informed consent, and answered the first survey question after consent, but discontinued the survey before completing the questionnaire. S6 Appendix: Specifications tests. Note: Standard errors in parentheses. * p < 0.10, ** p < 0.05, *** p < 0.01. S7 Appendix: DCE Results. Table S7.A: Random-intercept logit model. Note: Coefficient; Standard errors in parentheses. * p < 0.10, ** p < 0.05, *** p < 0.01. Table S7.B: Random-intercept logit model with individual controls. Note: Coefficient; Standard errors in parentheses. * p < 0.10, ** p < 0.05, *** p < 0.01. S8 Appendix: Latent class logit models. Table S9.A: Model goodness of fit results. Note: AIC = Akaike information criterion; BIC = Bayesian [file pone.0345491.s001.docx]

Supporting Information for the paper “**Home over institution? New insights on older adults’ care preferences from a mixed-methods study in France**

# S1 Appendix: Literature review for disabled persons and LTC configurations

|  | Country | Study | Respondents | Attributes | Levels |
| --- | --- | --- | --- | --- | --- |
| Home Care + Nursing home | Netherland | Nieboer et al., 2010 | Population générale de 50 à 65 ans (N=1082)  4 hypothetical patient profiles : frail+ lives alone, frail + married, dementia+ lives alone , dementia+ married | Number of hours of care per week: | *4 h / 8 h /12 h /16 h* |
|  |  |  |  | Organized social activities | *Not available / 1 half-day per week / 2 half-days per week / 3 half-days per week* |
|  |  |  |  | Transportation services | *Available / not available* |
|  |  |  |  | Living situation | *Living independently at home / Apartment building in the proximity of care / Sheltered accommodation / Elderly or nursing home* |
|  |  |  |  | Who provide care | *Regular care provider / Varying care providers* |
|  |  |  |  | Individual preferences | *Standardized care / The content of care is determined individually* |
|  |  |  |  | Coordinated care services delivery | *Have to arrange a little / have to arrange a lot* |
|  |  |  |  | Punctuality | *Max 15 min waiting time / max 1h. Waiting time* |
|  |  |  |  | Waiting list in month | *Directly available / 4 months / 8 months / 12 months* |
|  |  |  |  | Co-payment per week | *No co-payment / 50€ / 100€ / 150€* |
|  | England | (Dixon et al., 2015b) | Patients en sortie d’hôpital vivant à domicile (N=77) | Location of care (soins médicaux en sortie d’hôpital) | *Home, hospital, outpatient, nursing home* |
|  |  |  |  | Frequency of care | *1/wk, 3/wk, 7/wk, 15/wk* |
|  |  |  |  | Principal carer | *Support worker/ nurse/ therapist (e.g., physio, social worker, doctor)* |
|  | Chine | Leng et al., 2024 | 2031 adults aged 50-70 | Type of caregivers | *Informal caregiver / formal CG* |
|  |  |  |  | Place of LTC | *Home / community / nursing institutions / medical institutions* |
|  |  |  |  | Contents of LTC | *Basic living care / medical care / hospice care* |
|  |  |  |  | Out-of-pocket payments (per day) | *$2.23 / $ 5.21 / $11.90* |
|  |  |  |  | Quality of life (QoL) (physical pain more or less relieved and the mood) | *Poor / medium / good* |
| Home Care | Danemark | (Amilon et al. 2020) | Population générale (+18 ans)  Avec scenario unique hypothétique : femme de 83 ans ayant des limitations physiques qui vit seule, avec deux enfants mais qui vivent loin | Cleaning | *1h every second week / 1h every week* |
|  |  |  |  | Choice in the meal delivery service | *No choice / Choice between different meal* |
|  |  |  |  | Time for socialising in connection with the cleaning | *No time for socialising / 15 min every second week* |
|  |  |  |  | Possible to buy additional services from the municipality | *Not possible / possible for a user fee* |
|  |  |  |  | Increase in household taxes ($/year) | *0, 93, 187, 377, 933, 1 866* |
|  | Netherland | De Bresser et al., 2022 | Internet-based household panel of approximately 5000 households that is representative of the non-institutionalized Dutch population | Monthly premium per person | *30€ / 60€ / 90€ / 120€* |
|  |  |  |  | Hours of domestic care per week | *0h / 2h / 5h* |
|  |  |  |  | Minutes of personal care per day | *0 min / 45 min / 60 min / 90 min* |
|  |  |  |  | Hours of personal supervision and company per week | *0h / 5h / 10h* |
|  |  |  |  | Monthly annuity for assisting devices or services | *0€ / 50€ /100€* |
|  |  |  |  | One-off budget for home adaptations | *0€ / 4000€ / 8000€* |
|  | Irland | Walsh et al., 2020 | 551 people living at home with moderate dementia | Communication with professionals | *Standardised / personalised* |
|  |  |  |  | Flexibility of service provision | *Low / high* |
|  |  |  |  | Number of (publically funded) home care hours (per week) | *10h / 15h / 20h* |
|  |  |  |  | Co-payment | *No co-payment / Means-tested co-payment / compulsory co-payment* |
|  |  |  |  | Additional taxation per year (for home care system) | *50€ / 100€ / 150€ / 200€ / 250€* |
|  | Australie | Kaambwa et al., 2015 | 87 consommateurs de LTC (PA) et 30 aidants | Choice of service provider(s) | *Single service provider / Multiple service providers / Multiple service providers and other individuals including family* |
|  |  |  |  | Budget management | *The individual (client) / An informal carer / The service provider* |
|  |  |  |  | Saving unused funds | *Save all unused funds / Save half unused funds / Not able to save unused funds* |
|  |  |  |  | Choice of (day-to-day) support/care workers (including relatives): | *All your support workers / Some of your support workers / None of your support workers* |
|  |  |  |  | Support worker flexibility (changing activities) | *Fully flexible / Partly flexible / Inflexible* |
|  |  |  |  | Level of contact with service coordinator | *High contact (monthly) / Medium contact (every three months) / Low contact (every six months)* |
|  | Germany | (Lehnert et al., 2018b) | Population générale entre 45-60 ans (N=1209) | Care time (per day) | *30, 60, 90, 120 min/day* |
|  |  |  |  | Range of services offered by the HCBS (home and community-based services) provider | *Standard (personal care + housekeeping services + supervision & assistance + medical services between 7h and 20h) VS Extended (Standard services are offered 24h + case manager)* |
|  |  |  |  | Quality of care | *Very high , high, satisfactory, sufficient (HCBS outpatient care providers are evaluated annually by a healthcare service – based on 49 important criteria for providing care)* |
|  |  |  |  | Different professional caregiver (per month) | *1-2 / 3-5 / 6-8* |
|  |  |  |  | Co-payment (per month) | *0€ / 300€ / 600€ / 900€* |
| Nursing home | Australia | Milte et al., 2018 - 2022 | Milte et al., 2018: Population : NH residents (N=126) or family proxies (N=416)  Milte et al., 2022: Population générale entre 30-60 ans et >60 ans (futurs consommateurs) (N=701) ; residents (N=126), caregivers (N=416) | How much time care staff able to spend with me ? | *Care staff are always/sometimes/rarely able to spend enough time attending to my individual needs* |
|  |  |  |  | Do the shared spaces of the aged care home as a whole make you feel ‘at home’? | *I feel very at home here / I feel at home here sometimes / rarely* |
|  |  |  |  | Does your own room here make you feel ‘at home’? | *I feel very at home in my room / I feel at home in my room sometimes / rarely* |
|  |  |  |  | Is there access to outside and gardens in this aged care home? | *I can get outside … whenever I want / sometimes / not easily* |
|  |  |  |  | How often does the aged care home offer me things to do that make me feel valued? | *Very often / sometimes / rarely* |
|  |  |  |  | How flexible is the aged care home with the care routines (e.g. when you get out of bed, shower, eat your meals) | *Very /little flexible: The aged care home is [very happy to /sometimes] change the times they provide help if I require it;*  *Not much flexibility: Care and assistance seems to occur when it most suits the aged care home* |

# S2 Appendix: Scenario and choice tasks presentation

After an introductory paragraph of the DCE, we asked respondents to make choices for themselves and to imagine themselves in a situation of heavy dependency.

*Introduction of the DCE*

In France, one in five people over the age of 85 is currently considered 'dependent', meaning that they need help with daily activities because of their age or health problems. You may one day have health problems that require daily assistance. We would like to know your preferences and expectations if this were to happen. To do this, we'll ask you to put yourself in a hypothetical situation where [Hypothetical scenario].

Four scenarios were randomly assigned, two for cognitive problems and two for physical problems, with examples of the repercussions on daily life:

- Cognitive problems: *“You're experiencing increasing memory lapses, difficulty orientating yourself in time or space or carrying out activities of daily living. For example, in the morning, you don't always remember to get dressed or wash up, and you can no longer prepare breakfast on your own. You also sometimes leave home without a purpose and get lost outside. Finally, you have recently started to behave aggressively towards relatives around you.”*
- Physical problems: *“You are now in a wheelchair with significant physical problems. You can no longer move around your home alone: you must be cared for to get from bed to chair. You can no longer dress yourself or wash yourself. However, you still have your wits about you.”*

This scenario was preceded by a description of the couple's situation at the time of the difficulties. For people not in a couple: “You are living alone in your own home”, and for people in a couple, two situations were randomly allocated: either “Your partner has died, you are now living alone in your own home” or “you're still living with your partner”.

50.4% of respondents were exposed to the scenario with physical difficulties and 49.6% were exposed to the scenario with cognitive difficulties. And 64.3% of respondents were exposed to the scenario where they were alone when making their choices, while 35.7% were still living with their partner.

**Figure S2 : Choice tasks presentation**


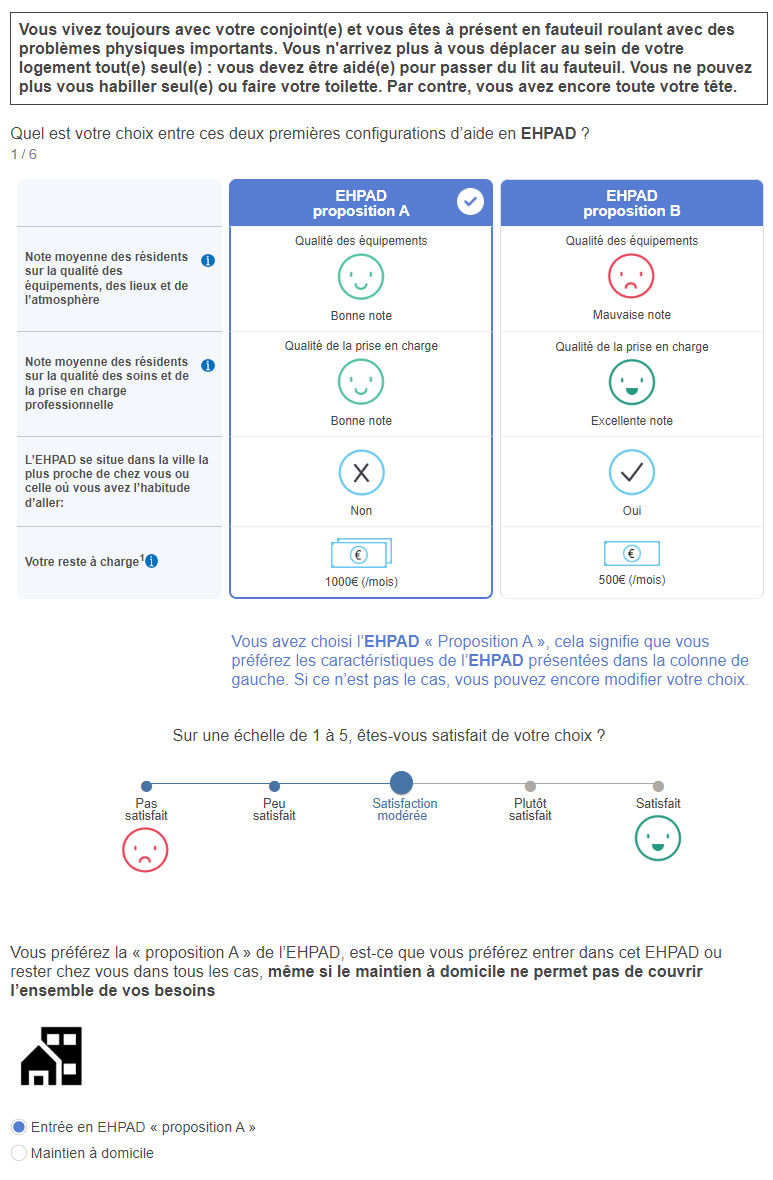


# S3 Appendix: Qualitative phase

1. Qualitative interviews for the selection of nursing-home attributes

To be included in the qualitative phase, people had to live in France near a survey site (3 selected areas: Eure-et-Loire, Paris and Isère), be over 60 years old, have no significant cognitive problems (i.e., be able to hold a conversation and not exhibit memory difficulties likely to compromise their ability to answer the interview questions), and be able to communicate in French. We chose to include people not yet affected by the loss of autonomy (in particular, the under-75s) because this population will be the following beneficiaries of autonomy policies (and their preferences and expectations will no doubt be different from those of today's dependents persons, due to a generation gap).

The interviews lasted an average of 45 minutes.

Of the 21 people interviewed, 15 were women and 6 men, with an average age of 82. Eight people are affected by physical problems (difficulty moving around and carrying objects), and one person is affected by cognitive problems (difficulty finding one's bearings in time and space). Eight were widowed, ten were in a couple and three were divorced, separated, or living alone.

**Table S3.A: Characteristics of people surveyed for the qualitative stage**

| Sex | Age | Marital status | Nb children | Rural / urban | Housing | Housing status | Identification |
| --- | --- | --- | --- | --- | --- | --- | --- |
| Female | 81 | Widow | 2 | Large town | Flat | Owner | PAF01 |
| Female | 75 | Couple | 2 | Rural | House | Owner | PAF02 |
| Female | 75 | Couple | 1 | Small town | House | Owner | PAF03 |
| Female | 84 | Widow | 1 | Small town | Flat | Owner | PAF04 |
| Male | 85 | Widowed | 1 | Small town | House | Owner | PAH05 |
| Female | 89 | Widow | 3 | Small town | House | Owner | PAF06 |
| Female | 86 | Widow | 1 | Small town | House | Owner | PAF07 |
| Male | 84 | Widow | 2 | Small city | House | Owner | PAH08 |
| Male + Female | 91 - 88 | Couple | 1 | Big city | Flat | Owner | PAH09  PAF18 |
| Female | 65 | Couple | 2 | Small city | House | Owner | PAF10 |
| Female | 89 | Divorced | 2 | Big city | Flat | Tenant | PAF11 |
| Female | 89 | Widowed | 2 | Big city | Flat | Owner | PAF12 |
| Female | 88 | Single | 0 | Big city | Flat | Tenant | PAF13 |
| Female | 75 | Couple | 2 | Very large city | Flat | Owner | PAF14 |
| Female | 92 | Couple | 3 | Very large city | Flat | Owner | PAF15 |
| Female | 76 | Couple | 2 | Very large city | Flat | Owner | PAF16 |
| Female | 74 | Couple | 0 | Very large city | Flat | Owner | PAF17 |
| Male | 74 | Widowed | 7 | Rural | House | Owner | PAH18 |
| Male | 84 | Couple | 6 | Rural | Flat | Tenant | PAH19 |
| Male | 73 | Separated | 1 | Big city | Flat | Owner | PAH20 |

The interviews took the form of a hybrid between life stories and semi-structured interviews. They began with general instructions about the preferences of respondents about staying at home or entering a nursing-home in case of disability.

Interviews begin

Given that by 2050, more than a quarter of the French population will be over 65, the XXX Chair brings together economists working on the challenges of this demographic transition. We're looking to see what public policies and care organization structures will enable the elderly to live well in the years gained by rising life expectancy. More specifically, we're looking to provide new responses to the needs of people losing their autonomy, which are more in line with their preferences. To this end, we will be launching a major national survey in January. In order to finalize this survey, we need to better understand your views and preferences.

The interview is recorded and will be transcribed in full, but will only be used by our research team with anonymized content and for research purposes.

[Information on consent form, signature, collection of ID number, information on data sharing].

**The purpose of this interview is to better understand your expectations and preferences regarding how you will age. To begin with, could you tell me where you would like to grow old if you were losing your independence, or even if you were dependent (at home, in a nursing home) and who would help you (your relatives, professionals)?**

I'll let you speak as freely as possible, and then I'll step in to ask for details, clarifications or to go into subjects you haven't spontaneously addressed. The interview should last about 1 hour if that suits you. Is it good for you?

Interviews end

-Thank the participant

-Mention the results release (website)

-Give contact details to be contacted in case of questions or problems

**Table S3.B: Interview grid**

| Categories | Subcategories and sample reminder | Check list |
| --- | --- | --- |
| Typical socio-demographic characteristics | Sex |  |
|  | Age |  |
|  | Socio-professional groups / Diplom / Career path |  |
|  | Marital status (Spouse's age ; Spouse's state of health) |  |
|  | Number of children (Relationship with children; Geographical distance) |  |
|  | Parents still alive |  |
|  | Income, pensions |  |
|  | Address; rural/urban area |  |
| Housing features | Housing vs flat |  |
|  | Owner vs tenant |  |
|  | Number of people living in the house |  |
|  | Housing adapted to the disabled |  |
|  | Size of dwelling (need for special maintenance, etc.) |  |
|  | Length of residence |  |
| Preference for home-care vs institutional care | Images / perceptions of EHPADS (public, vs. private?) |  |
|  | Visits to EHPAD (regular?) |  |
|  | ***If preference for home-care:*** |  |
|  | Reasons for attachment to home? What does home mean? |  |
|  | Under what circumstances would you agree to enter a nursing home? Why refuse entry to a nursing home? |  |
|  | Could this choice change with the nature of the difficulties (e.g. cognitive difficulties)? |  |
|  | ***If preference for nursing-home care:*** |  |
|  | Why do you prefer to live in a nursing home? |  |
|  | What does a nursing home offer you that a home does not? |  |
|  | **Interaction between preferences and family structure** |  |
|  | Does your choice depend on the potential involvement of your relatives? |  |
|  | Would it be the same if you didn't have any relatives or a spouse who could help you (or if, on the contrary, you could mobilize some of the people around you)? |  |
|  | Which relatives could you mobilize, and would you like to mobilize them? |  |
|  | Do you think it's normal for your relative to help you if you need it? |  |
| Home features (potential attributes to be tested) | **Professional care** |  |
|  | Would you like professionals to come and help you if you need it, and for what type of help (ADL / IADL / housekeeping/ coordination and administrative procedures ...)? |  |
|  | What type of professionals: care assistants, nurses, housekeepers? |  |
|  | What do you expect most from professionals: that they are always the same, that they are competent in what they do, that they are on time, that you can trust them, that they are always the same (no turnover), that they are close to you and that you can talk to them about your daily life, etc.? |  |
|  | Standardized care vs adaptation to needs, flexinility of professional carers |  |
|  | Amount of hours? |  |
|  | Would you be willing to pay for more professional help? |  |
|  | Help in other aspects of your life: accompany you to medical appointments, keep you company… |  |
|  | **Others home-care services** |  |
|  | Technical support (alarm …) |  |
|  | Home adaptation solutions |  |
|  | Transportation services |  |
|  | Social activities |  |
|  | Meal delivery |  |
|  | Waiting list / time for the home-care services |  |
|  | **Informal care role** (number of care per week, daily care?) |  |
|  | Type of informal care (personal care, homework help, companionship) |  |
|  | Remuneration of informal carers? |  |
|  | Willingness to pay for home-care services |  |
| Nursing-home features (potential attributes to be tested) | Size of nursing home |  |
|  | Public or private? |  |
|  | Characteristics of the others residents |  |
|  |  |  |
|  | **Nursing-home equipment** |  |
|  | Shared spaces and home room (feel at home? / size) |  |
|  | Bring your own furniture |  |
|  | Pets allowed |  |
|  | Garden access |  |
|  | Quality of food |  |
|  | Social activities in the nursing home |  |
|  | **Professional care** |  |
|  | Time spend for each resident |  |
|  | Flexibility in the care routine and adaptability to each need |  |
|  | Waiting list / time to enter the nursing home |  |
|  | Informal care (and family) role and consideration in the nursing home organization (visits time and rules) |  |
|  | Willingness to pay for nursing-home entry |  |

**Table S3.C: Thematic tree of interviewees' expectations for nursing homes**

| **Thematic axis** | **Thematic sub-axis** | **Verbatim**^[[1]](#footnote-1)^ |
| --- | --- | --- |
| **Equipment, activities and organization of nursing home that allow to ‘feel like home’** | Equipment’s features, particularly the bedrooms (having a large bedroom, being able to bring some of your own furniture), the garden and shared areas, to make people feel at home. | *“I think I'd be happy, I've visited the apartments, the rooms are very spacious’’. (PAH20)*  *“My mother-in-law, her nursing home, she had a flat, really, three rooms, (...) she had a living room, a kitchen, a bedroom, a bathroom, a terrace, it was great! She had all her own furniture, it was like being at home” (PAF17)*  *“Her bedroom was ridiculous, she didn't have a dressing cupboard, it's really shameful.’’ (PAF07)* |
|  | Activities, visits from health professionals and interaction with residents to break the isolation and restore a social life | *“What I noticed about her was that in the last few years that she was at home, the visits were mainly from the nurses and other health professionals and her immediate family. She didn't have much of a social life outside. In her last years in a care home, she was lucky enough to have a nursing home where she was quite well looked after. She had physiotherapy, exercise, lectures, and music occasionally. She came down for her meals. That gave her a social life that was more open than the social life she had before. So I think that's quite good.” (PAF14)*  *« [Researcher: do you see yourself in a nursing home?] Yes, no problem. Because at home, I feel very alone... I like being with people. » (PAH20)*  *“I wouldn't stay at home on my own. I think I'd do very well in a decent nursing home, where I could be comfortable and find people to talk to.” (PAF18)* |
|  | Quality of food and menu choice | *“In most nursing homes, people are served meals by the plate, e.g., everyone has the same plate. There is no choice, everyone gets the same portion.” (PAF15)*  *“My cooking will be done. My laundry will be done. Because at home, I'm the one who has to do everything.” (PAF03)* |
| **Nursing home to ensure continuous care, while respecting the individuality of each resident** | Receive round-the-clock care and feel safer than at home | *“If it gets too heavy and I need care or help with washing, I would prefer to go to a nursing home.” (PAF01)*  *“At home, I'm afraid at night. And if anything happens to me, no one is there. If I need oxygen or an injection, there's no one there.” (PAF15)* |
|  | Quality of professionals care | *“There were nurses who were more or less gentle. And turnover meant that less experienced people were doing replacements.” (PAF14)* |
|  | Fear that the rules of collective living will infringe on the freedom of each resident | *“If that happens, I hope we'll be able to find a place in a nursing home where human rules have priority over hygiene and rigid rules. Some are very good, but there are too many where we are subjected to rules that are abusive and where we are in a submissive situation where we have no say, no dialogue, we can't say anything. For example, some people have no choice when it comes to food. The quantity is the same for everyone. Some are even hungrier, others less so.” (PA F15)* |
|  | A desire for personalized care and support tailored to each resident, based on dialogue with professionals | *“I know a person in nursing home who is a football fan, but he can't watch the big games on his television because he's systematically given a sleeping pill with his dinner at 7am and he's asleep by 8am.” (PAF15)* |
| **Geographical proximity of the nursing home** | Choosing a nursing home in your neighborhood/town/village to keep your bearings and friendships | *“Oh, I know the one who cooks lunch, for example, my granddaughter went to school with her, you're from such and such a village, etc.'. We're already family, we're in the same environment'.” (PAF15)* |
|  | Choose a nursing home further away to be closer to your children or family and make it easier for them to visit you | *“If I leave, I'll be closer to my sister, and we can go together.” (PAF04)* |
| **Affordability of nursing homes** | Difficulty paying for nursing home with one's pension | *“With my savings, I could go, but it depends on how long I stay.” (PAF04)* |
|  | Not wanting children to contribute to the cost of nursing home (penalize them) or children don't want to / can't contribute | *“I had 1,200 euros to pay. They're asking for my children's tax returns. I have two boys, one earns well, the other not so well, he wasn't taxable. So we divide by two. So my son had to give 500 euros and I had to give 500 euros. My son said to me, "Wait, I've got three children. 500, even if he's my father, it's not possible. And my wife, would she agree to pay? Well, no. So I say, well, well, well. So we don't talk about it anymore'.” (PAF01).*  *“Also, if I go into a nursing home, it may come out of the estate when I die. It has to be a collective decision. Not all the children are ready to participate either.” (PAF10)* |

1. Think-aloud interviews

Once the attributes had been identified, an initial version of the DCE was tested on four people. These 4 pre-pilot cognitive interviews used think-aloud methodology to check that the attributes were clear and well understood and that the proposed levels made sense to the respondents. During these interviews, respondents were asked to describe their thoughts about the attributes and levels identified in the experiment, as well as the choices they were making (Ryan et al., 2009). We had changed the attributes related to the quality. Whereas in a first version of the DCE, we had selected the dimensions that seemed to emerge most from the interviews and excluded the others, the think-aloud interviews revealed that there was no consensus on the dimensions selected, and that some important dimensions were not mentioned. Given that the important dimensions are manifold and vary from person to person, we have decided to create two overall quality score. Before the DCE module, we asked respondents to select the three dimensions that seemed most important to them for each quality. These changes were made iteratively throughout the DCE testing phase: the questionnaire was modified based on feedback from the pre-pilot interviews, and then retested.

# S4 Appendix: Experimental design

**Table S4.A: Matrice correlation for nursing-home design**

|  | Rating: Equipment | Rating : care | Localisation | Out-of pocket cost |
| --- | --- | --- | --- | --- |
| Rating: equipment | 1.0000 |  |  |  |
| Rating : care | -0.0924 | 1.00000 |  |  |
| Localisation | -0.1098 | -0.1614 | 1.0000 |  |
| Out-of pocket cost | -0.0451 | -0.0948 | -0.1245 | 1.0000 |

**Table S4.B: 36 choice sets of nursing home DCE**

| Rating: Equipment | Rating : Prof. care | Localisation | Out-of pocket cost | Choice set | Alternative | Utility |
| --- | --- | --- | --- | --- | --- | --- |
| Block 1 | | | | | | |
| Excellent rating | Excellent rating | Yes | 1 000 € | 1 | 1 | 4.884 |
| Average rating | Good rating | No | 500 € | 1 | 2 | 2.885 |
| Good rating | Excellent rating | No | 2 000 € | 2 | 1 | 2.743 |
| Excellent rating | Poor rating | Yes | 1 000 € | 2 | 2 | 2.328 |
| Average rating | Excellent rating | Yes | 2 500 € | 3 | 1 | 2.069 |
| Poor rating | Average rating | No | 500 € | 3 | 2 | 1.068 |
| Excellent rating | Good rating | No | 2 000 € | 4 | 1 | 2.89 |
| Good rating | Excellent rating | Yes | 1 000 € | 4 | 2 | 4.617 |
| Good rating | Excellent rating | No | 1 000 € | 5 | 1 | 3.756 |
| Excellent rating | Poor rating | Yes | 500 € | 5 | 2 | 2.677 |
| Average rating | Average rating | Yes | 3 000 € | 6 | 1 | -.375 |
| Poor rating | Poor rating | No | 2 000 € | 6 | 2 | -1.362 |
| Block 2 | | | | | | |
| Excellent rating | Poor rating | Yes | 2 000 € | 7 | 1 | 1.315 |
| Poor rating | Good rating | Yes | 3 000 € | 7 | 2 | .544 |
| Average rating | Poor rating | Yes | 500 € | 8 | 1 | 1.31 |
| Excellent rating | Average rating | No | 2 500 € | 8 | 2 | 1.087 |
| Excellent rating | Good rating | Yes | 3 000 € | 9 | 1 | 2.36 |
| Good rating | Average rating | No | 500 € | 9 | 2 | 2.617 |
| Average rating | Good rating | No | 2 500 € | 10 | 1 | 1.088 |
| Poor rating | Excellent rating | Yes | 2 000 € | 10 | 2 | 2.055 |
| Good rating | Good rating | Yes | 2 000 € | 11 | 1 | 3.484 |
| Excellent rating | Excellent rating | No | 500 € | 11 | 2 | 4.372 |
| Good rating | Excellent rating | No | 3 000 € | 12 | 1 | 1.352 |
| Average rating | Average rating | Yes | 1 000 € | 12 | 2 | 2.029 |
| Block 3 | | | | | | |
| Average rating | Good rating | Yes | 2 000 € | 13 | 1 | 2.384 |
| Excellent rating | Excellent rating | No | 500 € | 13 | 2 | 4.372 |
| Average rating | Excellent rating | No | 1 000 € | 14 | 1 | 2.656 |
| Good rating | Average rating | Yes | 2 500 € | 14 | 2 | 1.681 |
| Poor rating | Good rating | Yes | 2 500 € | 15 | 1 | 1.5 |
| Excellent rating | Average rating | No | 3 000 € | 15 | 2 | .131 |
| Good rating | Good rating | No | 1 000 € | 16 | 1 | 3.636 |
| Poor rating | Excellent rating | Yes | 500 € | 16 | 2 | 3.417 |
| Good rating | Poor rating | Yes | 3 000 € | 17 | 1 | -.343 |
| Poor rating | Good rating | No | 2 500 € | 17 | 2 | .639 |
| Average rating | Average rating | Yes | 2 000 € | 18 | 1 | 1.016 |
| Good rating | Poor rating | No | 2 500 € | 18 | 2 | -.248 |

# S5 Appendix: Data sample

**Table S5.A: Age group target quotas**

|  | 60-64years | 65-69years | 70-74years | 75-79years |
| --- | --- | --- | --- | --- |
| **60-80years** | 0,2885 | 0,2680 | 0,2545 | 0,1887 |

*Eurostat (2023)*

**Table S5.B: Regions target quotas**

|  | Ile de France | Centre - Val de Loire | Bourgogne-Franche-Comté | Normandie | Hauts-de-France | Grand Est | Pays de la Loire | Bretagne | Nouvelle-Aquitaine | Occitanie | Auvergne-Rhône-Alpes | PACA | Corse | RUP FR* |
| --- | --- | --- | --- | --- | --- | --- | --- | --- | --- | --- | --- | --- | --- | --- |
| **60-80years** | 0,143 | 0,041 | 0,046 | 0,053 | 0,085 | 0,084 | 0,058 | 0,057 | 0,104 | 0,098 | 0,117 | 0,083 | 0,006 | 0,026 |

*Eurostat (2023)*

** « Régions Ultrapériphériques françaises » : Guadeloupe, Martinique, France Guyane, La Réunion, Mayotte, Saint-Martin*

**Table S5.C: Income target quotas**

|  | <€1,500 | €1,500-€2,000 | €2,000-€3,000 | €3,000+ |
| --- | --- | --- | --- | --- |
| **60-80years** | 0,250 | 0,250 | 0,300 | 0,2 |

*INSEE data* (admin data, public and private sector combined; no age criteria): 1st decile = 1440; 1st quartile=1680; Median=2095; 3rd quartile=2765; 9th decile=3765. Furthermore, according to the 2010 Wealth Survey, median salary levels are not significantly different between those under and over 50. However, salaries fall slightly from the age of 65.

**Table S5.D: Dropout Sample (N=950)**

| Variables | Measurement | N=2651 | % | N=950 | % | N=792 | % |
| --- | --- | --- | --- | --- | --- | --- | --- |
| **Respondents characteristics** | | Final Sample | | Dropout Sample | | Dropout Sample during the DCE module | |
| Sex | Female | 1393 | 52.6 | 570 | 60.00 | 470 | 59.34 |
|  | Male | 1258 | 47.5 | 380 | 40.00 | 322 | 40.66 |
| Age | 60-64 | 773 | 29.2 | 256 | 26.95 | 216 | 27.27 |
|  | 65-69 | 704 | 26.6 | 251 | 26.42 | 198 | 25.00 |
|  | 70-74 | 580 | 21.9 | 200 | 21.05 | 171 | 21.59 |
|  | 75+ | 594 | 22.4 | 243 | 25.58 | 207 | 26.14 |
| Age | Mean (SD) | 69.12 | (5.9) | 69.69 | (6.40) | 69.82 | (6.51) |
| Average salaries / pension | Mean (SD) | 2314.28 | (1324) | 2166.6 | (1304) | 2209.36 | (1318) |
| Place of residence | Rural environment | 634 | 23.9 | 201 | 21.16 | 170 | 21.46 |
|  | Semi-urban or urban | 843 | 31.8 | 319 | 33.58 | 274 | 34.60 |
|  | Very dense urban | 1174 | 44.3 | 430 | 45.26 | 348 | 43.94 |
| In couple | Yes | 776 | 70.7 | 350 | 36.84 | 494 | 37.63 |
|  | No | 1875 | 29.3 | 600 | 63.16 | 298 | 62.37 |
| Number of children | 0 | 471 | 17.8 | 215 | 22.63 | 182 | 22.98 |
|  | 1 | 441 | 16.6 | 127 | 13.37 | 108 | 13.64 |
|  | 2 | 907 | 34.2 | 317 | 33.37 | 264 | 33.33 |
|  | 3+ | 832 | 31.4 | 191 | 30.63 | 238 | 30.05 |
| **Dropout time in the survey** | |  |  |  |  |  |  |
| Vision of NH vs home care / care repartition between formal and informal | |  |  | 109 | 11.47 |  |  |
| DCE (home-care scenarios) | |  |  | 625 | 65.79 | 625 | 78.91 |
| DCE (nursing-home scenarios) | |  |  | 167 | 17.58 | 167 | 21.09 |
| Questions after DCE (behavioral questions, economic-heath questions) | |  |  | 49 | 5.16 |  |  |

**The dropout sample includes individuals who met the inclusion criteria, provided informed consent, and answered the first survey question after consent, but discontinued the survey before completing the questionnaire.*

Compared to the final sample, dropouts were more frequently female (60.0% vs. 52.6%), slightly older on average (mean age 69.7 vs. 69.1 years), and had lower average income (mean monthly salary/pension €2167 vs. €2314). They were also somewhat less likely to live as a couple (63.2% vs. 70.7%). Differences in place of residence and number of children were modest. The subgroup who dropped out during the DCE module displayed similar patterns.

# S6 Appendix: Specifications tests

|  | (1) | (2) | (3) | (4) | (5) | (6) | (7) | (8) |
| --- | --- | --- | --- | --- | --- | --- | --- | --- |
| Fixed effects  (coefficients) | Rating & cost categorical | Rating & cost continuous | Rating & cost quadratique | Rating continuous & cost categorical | Rating quadra & cost categorical | Rating categorical & cost continuous | Rating categorical & cost quadra | Step coding |
| Equipment rating, atmosphere (reference: poor rating) |  |  |  |  |  |  |  |  |
| Medium rate | 0.399^***^ |  |  |  |  | 0.380^***^ | 0.362^***^ |  |
|  | (0.123) |  |  |  |  | (0.118) | (0.118) |  |
| Good rate | 1.333^***^ |  |  |  |  | 1.280^***^ | 1.268^***^ |  |
|  | (0.134) |  |  |  |  | (0.122) | (0.123) |  |
| Excellent rate | 1.648^***^ |  |  |  |  | 1.631^***^ | 1.677^***^ |  |
|  | (0.125) |  |  |  |  | (0.121) | (0.120) |  |
| Equipment rating, atmosphere (continue) |  | 0.531^***^ |  | 0.531^***^ |  |  |  |  |
|  |  | (0.036) |  | (0.037) |  |  |  |  |
| Equipment rating, atmosphere (quadratic) |  |  | 0.094^***^ |  | 0.090^***^ |  |  |  |
|  |  |  | (0.007) |  | (0.007) |  |  |  |
| Quality of equipment: medium vs poor |  |  |  |  |  |  |  | 0.399*** |
|  |  |  |  |  |  |  |  | (0.123) |
| Additional effect good vs medium |  |  |  |  |  |  |  | 0.933*** |
|  |  |  |  |  |  |  |  | (0.105) |
| Additional effect excellent vs good |  |  |  |  |  |  |  | 0.315*** |
|  |  |  |  |  |  |  |  | (0.096) |
| Quality of care and care professionals (reference: poor rating) |  |  |  |  |  |  |  |  |
| Medium rate | 1.031^***^ |  |  |  |  | 1.054^***^ | 1.100^***^ |  |
|  | (0.138) |  |  |  |  | (0.136) | (0.137) |  |
| Good rate | 2.011^***^ |  |  |  |  | 2.040^***^ | 2.052^***^ |  |
|  | (0.155) |  |  |  |  | (0.149) | (0.150) |  |
| Excellent rate | 2.225^***^ |  |  |  |  | 2.228^***^ | 2.246^***^ |  |
|  | (0.128) |  |  |  |  | (0.127) | (0.127) |  |
| Quality of care and care professionals (continue) |  | 0.706^***^ |  | 0.703^***^ |  |  |  |  |
|  |  | (0.036) |  | (0.037) |  |  |  |  |
|  |  |  |  |  |  |  |  |  |
| Quality of care and care professionals (quadra) |  |  | 0.123^***^ |  | 0.123^***^ |  |  |  |
|  |  |  | (0.007) |  | (0.007) |  |  |  |
| Quality of care and care professionals: medium vs poor |  |  |  |  |  |  |  | 1.031*** |
|  |  |  |  |  |  |  |  | (0.138) |
| Additional effect good vs medium |  |  |  |  |  |  |  | 0.980*** |
|  |  |  |  |  |  |  |  | (0.123) |
| Additional effect excellent vs good |  |  |  |  |  |  |  | 0.214* |
|  |  |  |  |  |  |  |  | (0.113) |
| Nursing home in proximity | 0.660^***^ | 0.506^***^ | 0.370^***^ | 0.518^***^ | 0.408^***^ | 0.630^***^ | 0.585^***^ | 0.660^***^ |
|  | (0.085) | (0.076) | (0.073) | (0.077) | (0.075) | (0.081) | (0.080) | (0.085) |
| Cost (reference: 500€) |  |  |  |  |  |  |  |  |
| 1000 | -0.415^***^ |  |  | -0.277^***^ | -0.155 |  |  |  |
|  | (0.113) |  |  | (0.104) | (0.104) |  |  |  |
| 2000 | -1.155^***^ |  |  | -1.070^***^ | -1.051^***^ |  |  |  |
|  | (0.118) |  |  | (0.115) | (0.114) |  |  |  |
| 2500 | -1.330^***^ |  |  | -1.177^***^ | -1.122^***^ |  |  |  |
|  | (0.131) |  |  | (0.125) | (0.124) |  |  |  |
| 3000 | -1.758^***^ |  |  | -1.517^***^ | -1.399^***^ |  |  |  |
|  | (0.150) |  |  | (0.142) | (0.142) |  |  |  |
| cost_ehpad500 |  | -0.311^***^ |  |  |  | -0.344^***^ |  |  |
|  |  | (0.023) |  |  |  | (0.024) |  |  |
|  |  |  |  |  |  |  |  |  |
| cost_ehpad_quadra500 |  |  | -0.043^***^ |  |  |  | -0.049^***^ |  |
|  |  |  | (0.003) |  |  |  | (0.004) |  |
|  |  |  |  |  |  |  |  |  |
| Out-of-pockets: 1000€ vs 500€ |  |  |  |  |  |  |  | -0.415*** |
|  |  |  |  |  |  |  |  | (0.113) |
| Additional effect 2000€ vs 1000€ |  |  |  |  |  |  |  | -0.740*** |
|  |  |  |  |  |  |  |  | (0.109) |
| Additional effect 2500€ vs 2000€ |  |  |  |  |  |  |  | -0.175 |
|  |  |  |  |  |  |  |  | (0.123) |
| Additional effect 3000€ vs 2500€ |  |  |  |  |  |  |  | -0.429*** |
|  |  |  |  |  |  |  |  | (0.144) |
| Log Likelihood | -1644.695 | -1668.065 | -1698.654 | -1666.005 | -1687.361 | -1645.872 | -1652.821 | -1644.695 |
| LR Chi2 | 1337.012 | 1290.272 | 1229.095 | 1294.392 | 1251.680 | 1334.657 | 1320.761 | 1337.012 |
| AIC | 3311.390 | 3344.131 | 3405.307 | 3346.010 | 3388.722 | 3307.745 | 3321.641 | 3311.390 |
| BIC | 3384.727 | 3370.799 | 3431.975 | 3392.679 | 3435.391 | 3361.081 | 3374.977 | 3384.727 |
| Likelihood-ratio test (ref: model 1) |  |  |  |  |  |  |  |  |
| LR chi2 |  | 46.74 | 85.33 | 107.92 | 42.62 | 2.35 | 16.25 |  |
| Prob > chi2 |  | 0.0000 | 0.0000 | 0.0000 | 0.0000 | 0.5021 | 0.0010 |  |

Coefficient; Standard errors in parentheses

^*^ *p* < 0.10, ^**^ *p* < 0.05, ^***^ *p* < 0.01

Likelihood ratio (LR) tests indicate that the specifications for models 2, 3, 4, 5 and 7 are significantly below the full categorical model (model 1) and its stepwise-coded variant (model 8). Only model 6, which uses categorical ratings and a continuous cost variable, does not significantly differ from model 1 in terms of fit (LR chi² = 2.35, p = 0.5021). Additionally, model 6 presents slightly better information criteria (AIC = 3307.745; BIC = 3361.081) than model 1 (AIC = 3311.390; BIC = 3384.727). However, model 6 assumes a linear cost effect, whereas our results suggest a discontinuity in disutility at the €2500 level—an effect clearly captured in model 8. This finding leads us to reject the linear cost assumption of model 6 and retain models that rely exclusively on categorical representations (models 1 and 8). Step coding (model 8) captures the incremental effect of each level compared to the previous one, allowing for a nuanced interpretation of preferences across attribute levels. Ultimately, models 1 and 8 yield identical predictions, as evidenced by their identical log-likelihood, AIC, and BIC values.

# S7 Appendix: DCE Results

**Table S7.A: Random-intercept logit model**

|  | Marginal effects | Standard errors |
| --- | --- | --- |
| **Dummy variable coding** | | |
| **Equipment rating, atmosphere (reference: poor rating)** |  |  |
| Average rating | 0.024^***^ | (0.006) |
| Good rating | 0.089^***^ | (0.08) |
| Excellent rating | 0.112^***^ | (0.007) |
| **Quality of care and care professionals (reference: poor rating)** |  |  |
| Average rating | 0.054^***^ | (0.007) |
| Good rating | 0.120^***^ | (0.009) |
| Excellent rating | 0.133^***^ | (0.007) |
| **Nursing home in proximity** | 0.040^***^ | (0.005) |
| **Out-of-pockets expenses** (reference: 500€) |  |  |
| 1000€ | -0.026^***^ | (0.008) |
| 2000€ | -0.073^***^ | (0.008) |
| 2500€ | -0.081^***^ | (0.008) |
| 3000€ | -0.105^***^ | (0.009) |
| **Step-wise coding (additional effect)** | | |
| **Quality of equipment** (additional effect) |  |  |
| Medium vs poor | 0.029^***^ | (0.008) |
| Good vs medium | 0.064^***^ | (0.006) |
| Excellent vs good | 0.020^***^ | (0.006) |
| **Quality of care and care professionals** (additional effect) |  |  |
| Medium vs poor | 0.067^***^ | (0.008) |
| Good vs medium | 0.066^***^ | (0.008) |
| Excellent vs good | 0.012^*^ | (0.007) |
| **Nursing home in proximity** | 0.040^***^ | (0.005) |
| **Out-of-pockets expenses** (additional effect) |  |  |
| 1000€ vs 500€ | -0.023^***^ | (0.007) |
| 2000€ vs 1000€ | -0.046^***^ | (0.007) |
| 2500€ vs 2000€ | -0.009 | (0.008) |
| 3000€ vs 2500€ | -0.028^***^ | (0.009) |
| **Relative importance attributes (share)** | | |
| **Quality of equipment** | 0.287 |  |
| **Quality of care and care professionals** | 0.341 |  |
| **Nursing home in proximity** | 0.102 |  |
| **Out-of-pockets expenses** | 0.269 |  |
| Observations | 15906 |  |

^*^ *p* < 0.10, ^**^ *p* < 0.05, ^***^ *p* < 0.01

**Table S7.B : Random-intercept logit model with individual controls**

|  | Marginal effects | Standard errors |
| --- | --- | --- |
| **Attributes** (dummy variable coding) | | |
| **Equipment rating, atmosphere (reference: poor rating)** |  |  |
| Average rating | 0.026*** | (0.007) |
| Good rating | 0.093*** | (0.009) |
| Excellent rating | 0.116*** | (0.008) |
| **Quality of care and care professionals (reference: poor rating)** |  |  |
| Average rating | 0.053*** | (0.008) |
| Good rating | 0.122*** | (0.009) |
| Excellent rating | 0.136*** | (0.007) |
| **Nursing home in proximity** | 0.043*** | (0.006) |
| **Out-of-pockets expenses** (reference: 500€) |  |  |
| 1000€ | -0.029*** | (0.008) |
| 2000€ | -0.076*** | (0.008) |
| 2500€ | -0.089*** | (0.009) |
| 3000€ | -0.109*** | (0.010) |
| **Additional variables** | | |
| Physical pb (vs cognitive) | 0.013 | (0.012) |
| Age (reference : 60-64 years old) |  |  |
| 65-69 | -0.015 | (0.018) |
| 70-74 | -0.021 | (0.019) |
| 75+ | -0.110*** | (0.017) |
| Live alone (DCE scenario) | 0.010 | (0.013) |
| Gender: female | -0.056*** | (0.014) |
| Average wage, pension | 0.000 | (0.000) |
| Real estate and financial assets (in ref <100 000€) |  |  |
| 100 000€-300 000€ | 0.001 | (0.017) |
| >100 000€ | -0.006 | (0.019) |
| Diploma (below high school diploma) |  |  |
| High School diploma | -0.004 | (0.017) |
| 1-4 years after high school | 0.005 | (0.016) |
| 5+ years after high school | 0.017 | (0.025) |
| Nursing home equipment in region*, N, (%) |  |  |
| Very high and medium levels | -0.002 | (0.016) |
| Low levels | -0.053** | (0.024) |
| Place of residence (reference: rural environment/ /) |  |  |
| Semi-urban | 0.004 | (0.019) |
| Medium urban | -0.018 | (0.021) |
| Very dense urban | 0.010 | (0.018) |
| Living in a house (vs. living in a flat) | -0.015 | (0.016) |
| Owner (vs tenant) | -0.031 | (0.019) |
| Nb of children | -0.007 | (0.006) |
| Past or present experience: caring for the elderly | 0.012 | (0.015) |
| Alzheimer's disease in the family | 0.026* | (0.015) |
| Residential facilities for dependent people version (vs « EHPAD ») | 0.033*** | (0.012) |
| Perceived health (reference: very good) |  |  |
| Good | -0.006 | (0.022) |
| Medium | 0.022 | (0.026) |
| Bad | 0.016 | (0.037) |
| Chronic pain | -0.006 | (0.014) |
| 75% chance of living to age 85 or over | 0.028** | (0.013) |
| Housing not at all adapted to the disabled | 0.017 | (0.013) |
| Having at least one child living nearby | 0.007 | (0.014) |
| Have helped other family members in the past, or help them often or very often (looking after grandchildren, etc.). | -0.014 | (0.014) |
| Help or have helped family members financially | 0.011 | (0.015) |
| Consider the possibility of one day becoming disabled | 0.076*** | (0.015) |
| Visiting a relative in a nursing home in the last 5 years (ref : yes) |  |  |
| No, since more than 5 years | -0.046*** | (0.015) |
| No, never | -0.072*** | (0.016) |
| Observations | 13746 |  |

Standard errors in parentheses

# * p < 0.10, ** p < 0.05, *** p < 0.01

# Note: Insee density index: 1. Very high level of nursing home facilities (> 145 places per 1,000 elderly people); 2. Medium level of facilities (between 120 and 145 places per 1,000); 3. Low level of facilities (< 120 places per 1,000).

# S8 Appendix: Latent class logit models

**Table S8.A: Model goodness of fit results**

| Number of classes | 2 | 3 | 4 | 5 |
| --- | --- | --- | --- | --- |
| Log Likelihood | -5901.648 | -5523.136 | -5454.925 | -5429.233 |
| Adjusted Pseudo R² |  |  |  |  |
| AIC | 11853.30 | 11122.27 | 11011.85 | 10986.47 |
| BIC | 12045.16 | 11413.90 | 11403.25 | 11477.63 |

*Notes : AIC = Akaike information criterion ; BIC = Bayesian information criterion.*

**Table S8.B: Latent class estimation**

|  | **Class 1** | | | **Class 2** | | **Class 3** | **Class 4** |
| --- | --- | --- | --- | --- | --- | --- | --- |
| Labelled  **Respondents characteristics** (% or SD) / Mean SD | Unconditional nursing home futures’ patients | | | Unconditional home-care futures’ patients | | Home-care futures’ patients but highly attributes sensitive | Standards-bound nursing home futures’ patients |
|  | 1 vs 2 | 1 vs 3 | 1 vs 4 | 2 vs 3 | 2 vs 4 | 3 vs 4 |  |
| Female N, (%) | 139 (45.42) | | | 839 (55.45) | | 273 (51.61) | 142 (46.86) |
|  | 0.000 | 0.085 | 0.004 | 0.000 | 0.006 | 0.876 | - |
| Mean Age (SD) | 68.83 (0.13) | | | 69.63 (0.06) | | 68.32 (0.10) | 68.29 (0.13) |
|  | 0.000 | 0.002 | 0.004 | 0.000 | 0.000 | 0.876 | - |
| Average salaries or pension | 2482.03 | | | 2266.62 | | 2298.96 | 2409.57 |
|  | 0.000 | 0.000 | 0.109 | 0.231 | 0.000 | 0.004 | - |
| Real estate and financial assets, N, (%) |  | | |  | |  |  |
| <100 000€ | 109 (40.07) | | | 506 (39.41) | | 169 (36.90) | 119 (42.96) |
| >= 100 000€ | 163 (59.93) | | | 778 (60.59) | | 289 (63.10) | 158 (57.04) |
|  | 0.838 | 0.393 | 0.493 | 0.344 | 0.274 | 0.103 | - |
| Diploma, N, (%) |  | | |  | |  |  |
| Less than/or high school diploma | 159 (51.96) | | | 875 (57.83) | | 290 (54.82) | 171 (56.44) |
| Higher than high School Diploma | 147 (48.04) | | | 638 (42.17) | | 239 (45.18) | 132 (43.56) |
|  | 0.059 | 0.425 | 0.268 | 0.228 | 0.653 | 0.652 | - |
| Diploma, N, (%) |  |  |  |  |  |  |  |
| Bachelor degree and less | 260 (84.97) | | | 1356 (89.62) | | 459 (86.77) | 270 (89.11) |
| More than a bachelor degree | 46 (15.03) | | | 157 (10.38) | | 70 (12.23) | 33 (12.97) |
|  | 0.018 | 0.469 | 0.128 | 0.072 | 0.789 | 0.324 | - |
| Number of children, mean (SD), ttest | 1.817 (1.32) | | | 1.965 (1.39) | | 2.074 (1.44) | 2.046 (1.37) |
|  | 0.000 | 0.000 | 0.000 | 0.000 | 0.036 | 0.413 | - |
| Having at least one child living nearby, N, (%) | 159 (51.96) | | | 792 (52.35) | | 283 (53.50) | 163 (53.80) |
|  | 0.902 | 0.668 | 0.650 | 0.648 | 0.645 | 0.934 | - |
| Live alone (DCE scenario) N (%) | 206 (67.32) | | | 970 (64.11) | | 335 (63.33) | 193 (63.70) |
|  | 0.284 | 0.244 | 0.347 | 0.747 | 0.891 | 0.576 | - |
| Scenario physical problems (vs cognitive problems) | 160 (52.29) | | | 741 (48.98) | | 261 (49.34) | 154 (50.83) |
|  | 0.291 | 0.411 | 0.718 | 0.886 | 0.557 | 0.680 | - |
| Nursing home equipment in region^[[2]](#footnote-2)^, N, (%) |  | | |  | |  |  |
| Very high and medium levels | 290 (94.77) | | | 1365 (90.22) | | 482 (91.12) | 278 (91.75) |
| Low levels | 16 (5.23) | | | 148 (9.78) | | 47 (8.88) | 25 (8.25) |
|  | 0.011 | 0.054 | 0.137 | 0.546 | 0.407 | 0.754 | - |
| Living in a very dense urban area, N, (%) | 150 (49.02) | | | 654 (43.23) | | 230 (43.48) | 140 (46.20) |
|  | 0.063 | 0.121 | 0.487 | 0.920 | 0.340 | 0.446 | - |
|  |  | | |  | |  |  |
| Living in a house (vs. living in a flat), N, (%) | 199 (65.03) | | | 1038 (68.61) | | 362 (68.43) | 193 (63.70) |
|  | 0.222 | 0.314 | 0.731 | 0.941 | 0.095 | 0.163 | - |
| Owner (vs tenant), N, (%) | 232 (75.82) | | | 1175 (77.66) | | 399 (75.43) | 213 (70.30) |
|  | 0.482 | 0.899 | 0.125 | 0.292 | 0.006 | 0.107 | - |
| Past or present experience: caring for the elderly | 236 (77.12) | | | 1028 (67.94) | | 347 (65.60) | 205 (67.66) |
|  | 0.001 | 0.000 | 0.009 | 0.545 | 0.922 | 0.321 | - |
| Alzheimer's disease in the family | 85 (27.78) | | | 290 (19.17) | | 114 (21.55) | 74 (24.42) |
|  | 0.001 | 0.042 | 0.346 | 0.236 | 0.037 | 0.340 | - |
|  |  | | |  | |  |  |
| Residential facilities for dependent people version (vs « EHPAD ») | 165 (53.92) | | | 712 (47.06) | | 257 (48.58) | 165 (54.46) |
|  | 0.028 | 0.137 | 0.895 | 0.546 | 0.019 | 0.103 | - |
| 75% chance of living to age 85 or over | 126 (41.18) | | | 597 (39.46) | | 203 (38.37) | 141 (46.53) |
|  | 0.575 | 0.425 | 0.183 | 0.660 | 0.022 | 0.021 | - |
| Housing not at all adapted to the disabled | 119 (38.89) | | | 562 (37.14) | | 200 (37.81) | 113 (37.29) |
|  | 0.565 | 0.757 | 0.685 | 0.786 | 0.961 | 0.883 | - |
| Have helped other family members in the past, or help them often or very often (looking after grandchildren, etc.). | 168 (54.90) | | | 803 (53.07) | | 262 (49.53) | 155 (51.16) |
|  | 0.559 | 0.134 | 0.354 | 0.160 | 0.542 | 0.651 | - |
| Consider the possibility of one day becoming disabled | 259 (84.64) | | | 1086 (71.78) | | 412 (77.88) | 248 (81.85) |
|  | 0.000 | 0.018 | 0.356 | 0.000 | 0.000 | 0.174 | - |
| Preference for private rather than public nursing homes | 137 (44.77) | | | 667 (44.08) | | 224 (42.34) | 137 (45.21) |
|  | 0.825 | 0.495 | 0.912 | 0.487 | 0.718 | 0.421 |  |
| Can count on children to help in case of disability |  | | |  | |  |  |
| Do not count on children or rarely | 287 | | | 1388 | | 501 | 283 |
| Yes, can count on children daily | 19 (6.21) | | | 125 (8.26) | | 28 (5.29) | 20 (6.60) |
|  | 0.225 | 0.580 | 0.844 | 0.026 | 0.330 | 0.436 | - |
| Visiting a relative in a nursing home in the last 5 years |  | | |  | |  |  |
| No | 150 (49.02) | | | 908 (60.01) | | 291 (55.01) | 153 (50.50) |
| Yes | 156 (50.98) | | | 605 (39.99) | | 238 (44.99) | 150 (49.50) |
|  | 0.000 | 0.095 | 0.716 | 0.044 | 0.002 | 0.209 | - |
| Perceived health |  | | |  | |  |  |
| Good or very good | 203 (66.34) | | | 481 (68.21) | | 186 (64.84) | 88 (29.04) |
| Medium or bad | 103 (33.66) | | | 1032 (31.79) | | 343 (35.16) | 215 (70.96) |
|  | 0.523 | 0.661 | 0.219 | 0.155 | 0.347 | 0.071 |  |
| Chronic pain | 149 (48.69) | | | 744 (49.17) | | 272 (51.42) | 146 (48.18) |
|  | 0.878 | 0.448 | 0.900 | 0.374 | 0.753 | 0.369 | - |
| Physical limitations | 98 (32.03) | | | 473 (31.26) | | 170 (32.14) | 93 (30.69) |
|  | 0.793 | 0.974 | 0.723 | 0.710 | 0.845 | 0.667 | - |

*Table note: Values are shown for each latent class as* n (%) *for categorical variables and* mean (SD) *for continuous variables. The columns labeled “1 vs 2”, “1 vs 3”, “1 vs 4”, “2 vs 3”, “2 vs 4”, and “3 vs 4” report pairwise p-values for differences between classes (χ² tests for categorical variables; two-sample t-tests for continuous variables). For example, the proportion of women was 45.42% in Class 1 and 55.45% in Class 2, and this difference was statistically significant (p < 0.001).*

**Table S8.C: Multinomial logit**

|  | **Classe 1 (ref classe 2)** | | **Classe 3  (ref classe 2)** | | **Classe 4  (ref classe 2)** | |
| --- | --- | --- | --- | --- | --- | --- |
|  | Marginal effects | Standard errors | Marginal effects | Standard errors | Marginal effects | Standard errors |
|  |  |  |  |  |  |  |
| Physical pb (vs cognitive) | 0,014 | 0,013 | -0,003 | 0,016 | 0,003 | 0,014 |
| Age (reference : 60-64 years old) |  |  |  |  |  |  |
| 65-69 | -0,005 | 0,019 | -0,039* | 0,023 | 0,003 | 0,020 |
| 70-74 | 0,001 | 0,020 | -0,056 | 0,024 | -0,004 | 0,021 |
| 75+ | -0,046** | 0,018 | -0,087 | 0,023 | -0,061*** | 0,018 |
| Live alone (DCE scenario) | 0,008 | 0,014 | 0,003 | 0,017 | 0,000 | 0,015 |
| Gender: female | -0,038 | 0,015 | -0,006 | 0,018 | -0,024 | 0,015 |
| Average wage, pension | 0,000 | 0,000 | 0,000 | 0,000 | 0,000 | 0,000 |
| Real estate and financial assets (in ref <100 000€) |  |  |  |  |  |  |
| >100 000€ | -0,025 | 0,017 | 0,035 | 0,021 | -0,005 | 0,018 |
| Diploma (reference: High School Diploma or less) |  |  |  |  |  |  |
| Higher than High School Diploma | 0,014 | 0,015 | 0,024 | 0,018 | -0,010 | 0,015 |
| Nursing home equipment in region^7^ (very high levels in ref) |  |  |  |  |  |  |
| Medium level | -0,007 | 0,018 | 0,013 | 0,020 | 0,005 | 0,018 |
| Low level | -0,067*** | 0,023 | 0,006 | 0,033 | -0,005 | 0,028 |
| Place of residence (reference: rural environment/ Semi-urban/ Medium urban) |  |  |  |  |  |  |
| Very dense urban | 0,018 | 0,015 | 0,002 | 0,018 | -0,002 | 0,015 |
| Living in a house (vs. living in a flat) | -0,005 | 0,017 | 0,022 | 0,021 | -0,018 | 0,017 |
| Owner (vs tenant) | 0,007 | 0,020 | -0,025 | 0,025 | -0,039* | 0,021 |
| Nb of children | -0,013** | 0,006 | 0,009 | 0,007 | 0,000 | 0,006 |
| Past or present experience: caring for the elderly | 0,008 | 0,016 | -0,002** | 0,019 | -0,001 | 0,016 |
| Alzheimer's disease in the family | 0,043 | 0,017 | -0,043 | 0,019 | -0,003* | 0,016 |
| Residential facilities for dependent people version (vs « EHPAD ») | 0,020** | 0,015 | -0,007 | 0,020 | 0,029** | 0,016 |
| 75% chance of living to age 85 or over | 0,028 | 0,013 | -0,018 | 0,016 | 0,034*** | 0,014 |
| Housing not at all adapted to the disabled | 0,007 | 0,014 | -0,004 | 0,017 | 0,047 | 0,014 |
| Having at least one child living nearby | 0,017 | 0,014 | 0,003 | 0,017 | 0,001 | 0,015 |
| Have helped other family members in the past, or help them often or very often (looking after grandchildren, etc.). | -0,003 | 0,015 | -0,007 | 0,018 | -0,017 | 0,015 |
| Help or have helped family members financially | -0,010 | 0,017 | -0,008 | 0,020 | 0,028* | 0,016 |
| Consider the possibility of one day becoming disabled | 0,055*** | 0,019 | 0,005 | 0,020 | 0,053*** | 0,019 |
| Can count on children to help in case of disability (Yes daily, vs: not at all, rarely and not daily) | 0,009 | 0,016 | -0,004 | 0,019 | 0,011 | 0,016 |
| Visiting a relative in a nursing home in the last 5 years (ref : no) | 0,030* | 0,017 | 0,001 | 0,019 | 0,033* | 0,017 |
| Good or very good perceived health (medium/ bad in reference) | -0,017 | 0,018 | -0,036* | 0,021 | 0,009 | 0,019 |
| Chronic pain | -0,005 | 0,016 | 0,006 | 0,019 | -0,017 | 0,016 |
| Physical limitations | 0,002 | 0,017 | -0,012 | 0,021 | 0,010 | 0,018 |
| Constant | -2,218*** | 0.453 | -1,050*** | 0,368 | -2,067*** | 0,437 |
| Observations | 2291 |  |  |  |  |  |

*Standard errors in parentheses*

*^*^ p < 0.10, ^**^ p < 0.05, ^***^ p < 0.01*

1. The verbatims were translated by the authors from French [↑](#footnote-ref-1)
2. Insee density index: 1. Very high level of nursing home facilities (> 145 places per 1,000 elderly people); 2. Medium level of facilities (between 120 and 145 places per 1,000); 3. Low level of facilities (< 120 places per 1,000). [↑](#footnote-ref-2)
