# Supplementary material for: Home over institution? New insights on older adults’ care preferences from a mixed-methods study in France
Source: PLoS One. 2026 Mar 24;21(3):e0345491. doi: 10.1371/journal.pone.0345491 (PMC13012468; doi:10.1371/journal.pone.0345491)
Supplement: S2 File — (PDF) [file pone.0345491.s002.pdf]

Variable Values

Value		Label
X02	1	Male
	2	Female
	3	Does not wish to comment
X01BIS	1	60-64
	2	65-69
	3	70-74
	4	75-79
	5	80
AGGLO	1	less than 2,000 (1)
	2	2,000 to 20,000 (2)
	3	20,000 to 100,000 (3)
	4	over 100,000 (4)
	5	Paris metropolitan area (5)
AREA_FR13	1	1) Ile-de-France (1)
	2	2) Centre-Val de Loire (2)
	3	3) Burgundy/Franche-Comté (3)
	4	4) Normandy (Upper and Lower) (4)
	5	5) Hauts-de-France (5)
	6	6) Grand Est (6)
	7	7) Pays de la Loire (7)
	8	8) Brittany (8)
	9	9) Nouvelle-Aquitaine (9)
	10	10) Occitanie (10)
	11	11) Auvergne/Rhône-Alpes (11)
	12	12) Provence-Alpes-Côte d'Azur (12)
	13	13) Corsica (13)
CSP_DETAILED	1	1 - Business owner/self-employed (farmer + craftsman, shopkeeper + business owner)
	2	2 - Executives
	3	3 - Intermediate professions
	4	4 – Employees
	5	5 - Manual workers
	6	6 - Not applicable
CSP_3	1	1 – CSP +
	2	2 – CSP -
	3	3 - Not in employment
D05	1	Less than €600
	2	From €600 to less than €800
	3	From €800 to less than €1,000
	4	From €1,000 to less than €1,200
	5	From €1,200 to less than €1,500
	6	From €1,500 to less than €1,800
	7	From €1,800 to less than €2,000
	8	From €2,000 to less than €2,500
	9	From €2,500 to less than €3,000
	10	From €3,000 to less than €4,000
	11	From €4,000 to less than €6,000

	12	Over €6,000
	13	I don't know
	14	I do not wish to answer
X05	1	Less than €600
	2	From €600 to less than €800
	3	From €800 to less than €1,000
	4	From €1,000 to less than €1,200
	5	From €1,200 to less than €1,500
	6	From €1,500 to less than €1,800
	7	From €1,800 to less than €2,000
	8	From €2,000 to less than €2,500
	9	From €2,500 to less than €3,000
	10	From €3,000 to less than €4,000
	11	From €4,000 to less than €6,000
	12	From €6,000 to less than €10,000
	13	Over €10,000
	14	I don't know
	15	I do not wish to answer
X06	1	Yes, with someone who lives in the household
	2	Yes, with someone who does not live in the dwelling
	3	No
X07	1	Single
	2	Cohabiting or civil partnership
	3	Married or remarried, including legally separated
	4	Widowed
	5	Divorced
X08	1	0
	2	1
	3	2
	4	3
	5	4
	6	5
	7	More than 5
proximity	1	Yes
	2	No
DOMEHPAD1_1	1	Home
	2	Nursing home*
	3	No difference
	4	I don't know
DOMEHPAD1_2	1	Home
	2	Nursing home
	3	No difference
	4	I don't know
DOMEHPAD1_3	1	Home
	2	Nursing home
	3	No difference
	4	I don't know
DOMEHPAD1_4	1	Home

	2	Nursing home
	3	No difference
	4	I don't know
DOMEHPAD1_5	1	Home
	2	Nursing home
	3	No difference
	4	I don't know
DOMEHPAD1_6	1	Home
	2	Nursing home
	3	No difference
	4	I don't know
DOMEHPAD2_1_DK_98	0	Not selected
	1	Selected
DOMEHPAD2_2_DK_98	0	Not selected
	1	Selected
DOMEHPAD2_3_DK_98	0	Not selected
	1	Selected
HIDVERSION	1	Version A: Nursing home
	2	Version B: Medical facility for dependent elderly people
DCE03	1	Yes
	2	No
DCE03BIS_1	0	Not selected
	1	Selected
DCE03BIS_2	0	Not selected
	1	Selected
DCE03BIS_3	0	Not selected
	1	Selected
DCE03BIS_4	0	Not selected
	1	Selected
DCE03BIS_5	0	Not selected
	1	Selected
DCE04_1	1	PUBLIC
	2	PRIVATE
A02	0	0 = Forward-thinking (anticipatory)
	1	1
	2	2
	3	3
	4	4
	5	5
	6	6
	7	7
	8	8
	9	9
	10	10 = Lives day to day (in procrastination)
A03	1	Yes, and I have taken steps (financial, administrative, practical, etc.) to deal with it
	2	Yes, but I have not yet made arrangements
	3	Yes, but I do not intend to make any arrangements
	4	No, I haven't considered it
	5	No, I don't want to hear about it.
A09	1	No
	2	With difficulty or only very occasionally
	3	Yes, but not every day
	4	Yes, daily
exp_carerPA	1	Yes
	2	No

A11	1	Yes, very often
	2	Yes, often
	3	Yes, occasionally
	4	No, never
A12	1	Yes
	2	No
A13	1	Every day
	2	Once or several times a week
	3	Several times a month
	4	Once a month
	5	At least once a year
	6	Never in the last 12 months
	7	Not applicable: no family
	8	I do not wish to answer
B01	1	Very good
	2	Good
	3	Average
	4	Poor
	5	Very poor
B02	1	Yes
	2	No
	3	Don't know
B03	1	Yes, severely limited
	2	Yes, limited, but not significantly
	3	No, not limited at all
	4	You don't know
B09	1	Yes
	2	No
	3	I don't know
B10	1	Yes
	2	No, for longer than that
	3	No, never
housing	1	Detached/farmhouse/detached house
	2	Semi-detached / terraced / grouped
	3	In a building with 2-9 dwellings
	4	In a building with more than 10 dwellings
	5	In precarious housing (trailer, caravan, etc.)
	6	Another type of dwelling - single person
	7	Residential care home for elderly dependents (EHPAD)
	8	An independent living facility (formerly known as "foyers-logements")
	9	A long-term care facility (USLD)
	10	Other medical-social or healthcare accommodation
	11	Another type of collective accommodation
C02	1	Fully (accessible rooms, adapted furniture, single-storey accommodation, etc.)
	2	Only partially
	3	Not at all (steps inside the dwelling or to enter the dwelling, etc.)
C03	1	1 "Do you live alone in this dwelling?"
	2	2
	3	3
	4	4
	5	5
	6	6
	7	7
	8	8

C04	1	Owner and you have finished repaying the loan
	2	Owner and you are still paying off the loan
	3	Tenant or subtenant (i.e. you have to pay rent, even if that rent is paid by someone else)
	4	Living rent-free, but with the possibility of paying service charges
	5	I don't know
car_essential	1	Yes
	2	No
C06	1	No qualifications
	2	CAP, BEP
	3	Baccalaureate
	4	DEUG, BTS, DUT, DEUST (two years of higher education)
	5	Bachelor's degree, professional bachelor's degree, BUT (Bac+3)
	6	Master's degree (Bac+4)
	7	Master's degree, advanced studies diploma (DEA), specialised higher education diploma (DESS), engineering diploma
	8	Doctorate, accreditation to supervise research (Bac+8)
C07	1	0 – €50,000
	2	€50,000 – €100,000
	3	€100,000 – €150,000
	4	€150,000 – €200,000
	5	€200,000 – €300,000
	6	€300,000 - €500,000
	7	Over €500,000
	8	I do not wish to answer
C08	1	Very difficult
	2	Difficult
	3	Somewhat difficult
	4	Quite difficult
	5	Easy
	6	Very easy
	7	I do not wish to answer
rural_urban	1	Rural
	2	Semi-urban
	3	Medium urban
	4	Very dense urban
HIDSCENARIO	1	Scenario 1: Strong cognitive _ lives alone
	2	Scenario 2: Strong cognitive _ does not live alone
	3	Scenario 3: Strong physical _ lives alone
	4	Scenario 4: Physically strong _ does not live alone
	5	Scenario 1bis: Strong cognitive _ lives alone
	6	Scenario 3a: Physically strong _ lives alone
region_density_nursing_homes	1	Very high equipment rate
	2	Average equipment rate
	3	Low equipment rate
ratingequip	1	Poor rating
	2	Average rating
	3	Good mark
	4	Very good rating
ratingsoins	1	Poor rating
	2	Average rating
	3	Good mark
	4	Very good rating
localis	0	No
	1	Nursing home nearby

cost_ehpad	1	500
	2	1000
	3	2000
	4	2,500
	5	3000
choiceEH_dummy	0	Nursing home 1
	1	Nursing home 2
pref_ehpad	0	Pref home care
	1	Pref nursing home

Variable Information

Variable	Position	Label
RESPID_BIS	1	Record number
X01	4	X01. BIRTH
X02	5	X02. SEX
X01BIS	7	X01. Age Recode
AGGLO	9	AGGLO - Size of the urban area
AREA_FR13	12	AREA.FR13 (Regions)
X04	13	X04. PCS
CSP_DETAILED	14	RECODE CSP DETAILED
D05	16	D05. SALARY
X05	18	X05. INCOME
X06	19	X06. COUPLE
X07	20	X07. MATRIMONIAL STATUS
X08	21	X08. NUMBER OF LIVING CHILDREN
enf_proximity	22	X09. CHILDREN NEARBY
DOMEHPAD1_1	24	DOMEHPAD1. HOME-CARE VERSUS NURSING HOME- The safest and most secure option
DOMEHPAD1_2	25	DOMEHPAD1. HOME-CARE VERSUS NURSING HOME - The cheapest option
DOMEHPAD1_3	26	DOMEHPAD1. HOME-CARE VERSUS NURSING HOME- The most reassuring, personalised and comprehensive care and support
DOMEHPAD1_4	27	DOMEHPAD1. HOME-CARE VERSUS NURSING HOME- The richest social life with plenty of leisure activities
DOMEHPAD1_5	28	DOMEHPAD1. HOME-CARE VERSUS NURSING HOME- The most satisfying living environment (cleanliness, quiet, feeling at home, etc.)
DOMEHPAD1_6	29	DOMEHPAD1. HOME-CARE VERSUS NURSING HOME- The greatest psychological burden for families (caregivers)
DOMEHPAD2_1_1	30	DOMEHPAD2.1. Preferred level of involvement in personal care (washing, dressing, eating or drinking, transferring from bed to chair, etc.) - Relatives
DOMEHPAD2_1_2	31	DOMEHPAD2.1. Preferred level of involvement in personal care (washing, dressing, eating or drinking, transferring from bed to chair, etc.) - Professional help
DOMEHPAD2_1_DK_98	32	DOMEHPAD2.1. CURSEURAIDE - Help with personal care (washing, dressing, eating or drinking, transferring from bed to chair, etc.) - I don't know
DOMEHPAD2_2_1	33	DOMEHPAD2.2. Preferred level of involvement - Help with housework, shopping, meals - Help from family and friends
DOMEHPAD2_2_2	34	DOMEHPAD2.2. Preferred level of involvement - Help with housework, shopping, meals - Professional help
DOMEHPAD2_2_DK_98	35	DOMEHPAD2.2. Preferred level of involvement - Help with housework, shopping, meals - I don't know
DOMEHPAD2_3_1	36	DOMEHPAD2.3. Preferred level of involvement - Help with administrative procedures, organising medical appointments, coordinating professionals, etc. - Help from friends and family
DOMEHPAD2_3_2	37	DOMEHPAD2.3. Preferred level of involvement - Assistance with administrative procedures, organising medical appointments, coordinating professionals, etc. - Professional assistance

DOMEHPAD2_3_DK_98	38	DOMEHPAD2.3. Preferred level of involvement - Assistance with administrative procedures, organising medical appointments, coordinating professionals, etc. - I don't know
DOMEHPAD3_1	39	DOMEHPAD3. QUALITEPROF - Punctuality: professionals are punctual (vs. professionals can arrive within a 2-hour time slot)
DOMEHPAD3_2	40	DOMEHPAD3. QUALITEPROF - Ability to adapt to needs: professionals adapt to the specific expectations of each patient (vs. standardised care)
DOMEHPAD3_3	41	DOMEHPAD3. QUALITEPROF - Empathy and good manners: warm relationships between professionals and patients, with particular attention paid to patients (vs. neutral relationships)
DOMEHPAD3_4	42	DOMEHPAD3. QUALITEPROF - Time spent on care: professionals take the time necessary to perform the tasks they need to perform and may take extra time to talk with the patient
DOMEHPAD3_5	43	DOMEHPAD3. QUALITEPROF - Trusting relationship: professionals are completely trustworthy (vs. sometimes it is necessary to monitor how long professionals spend on tasks and whether they are performing them correctly)
DOMEHPAD3_6	44	DOMEHPAD3. QUALITEPROF - Size of the team involved: 1 to 2 different professionals (vs. large team, you rarely see the same professionals)
DOMEHPAD3_7	45	DOMEHPAD3. QUALITEPROF - Stability of professionals: the professionals are very often the same (vs. professionals change all the time)
DOMEHPAD3_8	46	DOMEHPAD3. QUALITEPROF - I don't know
DOMEHPAD3_9	47	DOMEHPAD3. QUALITEPROF - I cannot identify the three most important criteria
HIDVERSION	48	Questionnaire version: Nursing homes or medical facilities for dependent elderly people
DOMEHPAD4_1	49	DOMEHPAD4. QUALITEEHPAD - Friendliness and atmosphere: friendly, welcoming and lively places
DOMEHPAD4_2	50	DOMEHPAD4. QUALITEEHPAD - Quality of common areas and rooms: spacious, bright common areas and rooms
DOMEHPAD4_3	51	DOMEHPAD4. QUALITEEHPAD - Access to gardens: access to gardens in common areas and rooms
DOMEHPAD4_4	52	DOMEHPAD4. QUALITEEHPAD - Feeling at home: feeling at home, possibility of bringing personal furniture into one's room
DOMEHPAD4_5	53	DOMEHPAD4. QUALITEEHPAD - Food quality: good food and choice of menus
DOMEHPAD4_6	54	DOMEHPAD4. QUALITEEHPAD - I don't know
DOMEHPAD4_7	55	DOMEHPAD4. QUALITEEHPAD - I cannot identify the three most important criteria
DCE01	329	DCE01. ReturnDCEdom
DCE02_1	330	DCE02. BackDCEdom2 - Assistance with home adaptations
DCE02_2	331	DCE02. Back to DCEdom2 - Other technical aids or telemedicine
DCE02_3	332	DCE02. Back to DCEdom2 - Possibility of having a general practitioner
DCE02_4	333	DCE02. ReturnDCEdom2 - Possibility of having nurses/caregivers
DCE02_5	334	DCE02. BackDCEdom2 - Possibility of using a taxi to get around
DCE02_6	33	DCE02. ReturnDCEdom2 - Other, please specify:
DCE02_6_OTHER	336	DCE02. ReturnDCEdom2 - Other, please specify:
DCE03	554	DCE03. ReturnDCEehpad
DCE03BIS_1	555	DCE03BIS. ReturnDCEehpad2 - Size of [VERSION]
DCE03BIS_2	556	DCE03BIS. RetourDCEehpad2 - Status of [VERSION]: public or private (non-profit associations or private commercial entities)
DCE03BIS_3	557	DCE03BIS. RetourDCEehpad2 - Possibility of bringing pets
DCE03BIS_4	558	DCE03BIS. BackDCEehpad2 - Other aspects relating to your interactions with professionals
DCE03BIS_5	559	DCE03BIS. BackDCEehpad2 - Other: please specify:
DCE03BIS_5_OTHER	560	DCE03bis. ReturnDCEehpad2 - Other: please specify:

DCE04_1	561	DCE04.1. STATUTEHPAD
A01	563	A01. RISK AVERSION
A02	564	A02. PROVISION
A03	565	A03. PROVISION FOR
probability_of_entering_a_nursing_home	573	A06. Nhprobability (probability of 0 to 100% of thinking about entering a nursing home one day)
A07	574	A07. FUTUR PREFERENCE
A09	580	A09. POTENTIAL INFORMAL CAREGIVERS
exp_aidantPA	581	A10. Carer experience
A11	582	A11. Mutual assistance
A12	583	A12. AIDEFIN Financial assistance
probability_of_survival85	590	A16. What is the percentage chance that you will live to be 85 or older?
B01	591	HEALTHSUBJ
B02	592	B02. Chronic diseases
B03	593	B03. Fonctional limitations
B09	594	B09. ALZHEIMER'S IN THE FAMILY
B10	600	B10. CONNAIEHPAD (EHPAD visit)
B11	601	B11. Subjective health
housing	602	PLACE OF RESIDENCE
C02B_1	603	C02B. ETAB - Due to your state of health or age
C02B_2	604	C02B. ETAB - Because your relatives could no longer help you
C02B_3	605	C02B. ETAB - Because you felt lonely (moving closer to your children, etc.)
C02B_4	606	C02B. ETAB - Because receiving professional help at home was too expensive
C02B_5	607	C02B. ETAB - For the services offered by the establishment
C02B_6	608	C02B. ETAB - To live with other people and no longer be alone
C02B_7	609	C02B. ETAB - Other reasons
C02	610	C02. HOUSING CHARACTERISTICS
C03	611	C03. Number of persons in the dwelling
C04	612	C04. HOUSING STATUS - OWNER
car_essential	613	C05. CAR
C06	614	C06. DIPLOMA

C07	615	C07. Value of financial and real estate assets
C08	616	C08. Financial difficulties
rural_urban	618	PLACE OF LIFE
scenario	620	DCE SCENARIO
region_density_ehpad	622	Rate of nursing home provision in regions
equipment rating	623	DCE- ATTRIBUTE 1: Equipment quality rating
ratingsoins	624	DCE- ATTRIBUTE 2: Quality rating of professionals
localis	625	DCE- ATTRIBUTE 3: Location of the nursing home
cost_ehpad	626	DCE- ATTRIBUTE 4: Cost of the nursing home
choiceEH_dummy [2]	627	Choice DCE EHPAD (EHPAD VERSION A or EHPAD VERSION B)
pref_ehpad	628	CHOICE BETWEEN HOME OR NURSING HOME
